# Heuristics and Learning Models for Dubins MinMax Traveling Salesman Problem

**DOI:** 10.3390/s23146432

**Published:** 2023-07-15

**Authors:** Abhishek Nayak, Sivakumar Rathinam

**Affiliations:** Mechanical Engineering, Texas A&M University, College Station, TX 77843, USA; nykabhishek@tamu.edu

**Keywords:** mission planning, unmanned vehicles, traveling salesman problem, Dubins vehicles, drones

## Abstract

This paper addresses a MinMax variant of the Dubins multiple traveling salesman problem (mTSP). This routing problem arises naturally in mission planning applications involving fixed-wing unmanned vehicles and ground robots. We first formulate the routing problem, referred to as the one-in-a-set Dubins mTSP problem (MD-GmTSP), as a mixed-integer linear program (MILP). We then develop heuristic-based search methods for the MD-GmTSP using tour construction algorithms to generate initial feasible solutions relatively fast and then improve on these solutions using variants of the variable neighborhood search (VNS) metaheuristic. Finally, we also explore a graph neural network to implicitly learn policies for the MD-GmTSP using a learning-based approach; specifically, we employ an S-sample batch reinforcement learning method on a shared graph neural network architecture and distributed policy networks to solve the MD-GMTSP. All the proposed algorithms are implemented on modified TSPLIB instances, and the performance of all the proposed algorithms is corroborated. The results show that learning based approaches work well for smaller sized instances, while the VNS based heuristics find the best solutions for larger instances.

## 1. Introduction

The multiple traveling salesman problem (mTSP) is a challenging optimization that naturally arises in various real-world routing and scheduling applications. It is a generalization of the well-known traveling salesman problem (TSP) [1,2], where given a set of *n* targets and *m* vehicles, the objective is to find a path for each vehicle, such that each target is visited at least once by some vehicle, and an objective which depends on the cost of the paths is minimized. The mTSP has many practical application, including but not limited to industrial robotics [3], transportation and delivery [4], monitoring and surveillance [5], disaster management [6,7], precision agriculture [8], search and rescue missions [9,10], multi-robot task allocation and scheduling [11], print press scheduling [12], satellite surveying networks [13], transportation planning [14], mission planning [15], co-operative planning for autonomous robots [16], and unmanned aerial vehicle planning [17].

**Figure 1:**
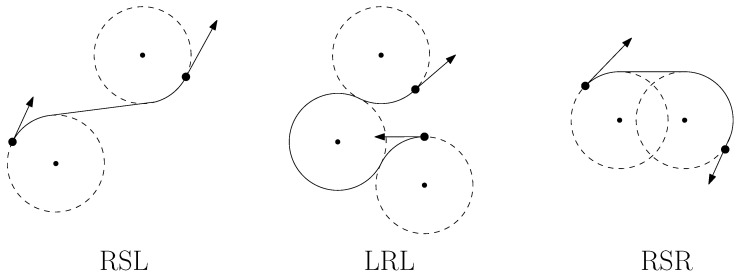
Examples of Dubins Paths.

The standard mTSP aims to find paths for multiple vehicles without taking into account the dynamics of the vehicles. However, some vehicles, like fixed-wing unmanned aerial vehicles and ground robots, have kinematic constraints that need to be considered while planning their paths. Specifically, these vehicles have yaw rate or turning radius constraints. To address this issue, L.E. Dubins [18] extensively studied the paths for curvature-constrained vehicles moving between two configurations. Dubins considered the model of a vehicle that moves at a constant speed $v_{0}$ on a 2D plane that cannot reverse and has a minimum turning radius ρ; this vehicle is also called the Dubins vehicle in the literature. The configuration of a Dubins vehicle is defined by its position and heading (x,y,θ), where *x* and *y* are the vehicle’s position coordinates on the plane, and θ is its heading angle. The motion of a Dubins vehicle can be fully defined using the following set of equations:

(1) $$\begin{array}{c} \dot{x}=v_{0}\cos\theta ,\;\dot{y}=v_{0}\sin\theta ,\;\dot{\theta }=\frac{v_{0}}{\rho }u,\;-1\leq u\leq 1 \end{array}$$

where *u* is the input to the vehicle and ρ is the minimum turning radius of the vehicle. Dubins proposed that in an obstacle-free environment, the shortest path between any two configurations on a 2D plane for a Dubins vehicle must be either the CCC type or the CSC type or a combination of sub-paths of these two types; here, *C* represents an arc of a circle with a radius of ρ, and *S* represents a straight line segment. These paths can be expressed as LSL, RSR, RSL, LSR, RLR, or LRL, where *L* and *R* indicate clockwise and counterclockwise turns of a *C* segment, respectively, as illustrated in Figure 1. When the vehicle is also capable of reversing, the shortest path problem was solved by Reeds and Shepp in [19]. Optimal solutions to these problems were also derived by Sussmann and Tang (1991) [20] as well as Boissonnat et al. (1994) [21], utilizing the Pontryagin maximum principle [22]. Tang and Özgüner (2005) [23] and Rathinam et al. (2007) [24] have further expanded upon Dubins’ research by applying it to routing problems involving car-like mobile robots or fixed-wing aerial vehicles that maintain a constant forward speed and adhere to curvature constraints while making turns.

The vehicle routing problem for a Dubins vehicle is called the Dubins traveling salesman problem (DTSP) [25]. In the DTSP, the challenge is not only to find the optimal order of the targets to visit but also to determine suitable heading angles for the vehicle when visiting the targets. A feasible solution to the DTSP involves a curved path where the radius of curvature at any point along the path is at least equal to the turning radius ρ≥0, and each target is visited at least once. Since the problem involves both discrete and continuous decision variables, with the target order being discrete and the heading angles being continuous, finding an optimal solution to the DTSP is an NP-hard problem, as shown by Jerome Le Ny et al. [26]. Currently, no exact algorithm exists in the literature that can find an optimal solution to the DTSP. However, over the last two decades, several heuristics and approximation algorithms have been developed to find feasible solutions for the DTSP [23,24,27,28,29,30,31,32,33,34].

**Figure 2:**
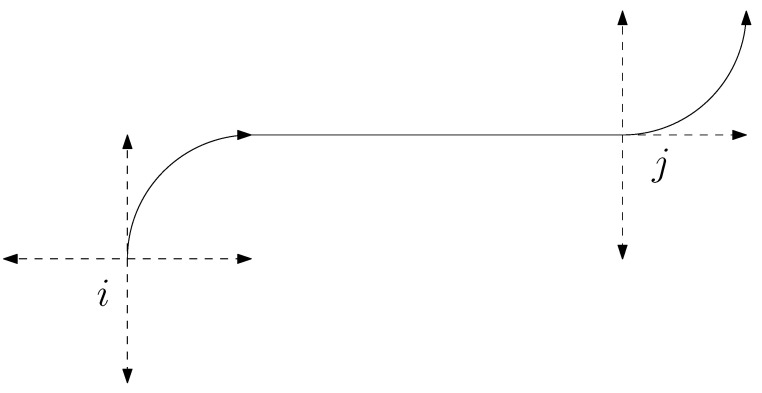
Dubins path between targets with discretized heading angles.

One popular approach to solving the DTSP, as discussed in several studies [33,35,36,37,38], involves sampling the interval [0,2π] into *k* candidate headings at each target and posing the resulting problem as a generalized traveling salesman problem (GTSP) formulation. In GTSP (also known as the one-in-a-set TSP), given *n* nodes partitioned into *k* mutually exclusive sets, the goal is to compute the shortest path that contains exactly one node from each of the *k* sets. GTSP is an NP-hard problem since it reduces to the TSP when each cluster contains only one node. DTSP can be reduced to a one-in-a-TSP by forming *n* mutually exclusive sets consisting of *k* vehicle configurations corresponding to *k* heading angles at each of the *n* targets. Increasing the angle discretizations at each target for the one-in-a-set TSP variant results in solutions that get close to the DTSP optimum. This approach provides a natural way to find a good feasible solution to the DTSP problem, as noted in previous studies [36]. Figure 2 illustrates the discretization of heading angles at a target for the DTSP.

In this work, we explore the MinMax variant of the mTSP problem for a Dubins-like vehicle by formulating it as a one-in-a-set TSP, called the MinMax Dubins generalized multiple traveling salesman problem (MD-GmTSP) and present three different approaches to solve it. The MinMax variant is an important problem to study as it directly relates the objective to the mission completion time for visiting all the targets by a team of vehicles. This variant is relevant for real-world applications as it accounts for the worst-case scenario in situations where *m* homogeneous vehicles are assigned travel paths by accounting for limitations on load/delivery capacities or refueling constraints.

### Contributions of This Article

- We formulate a mixed-integer linear program for the MD-GmTSP (Section 3).
- We develop heuristics to generate feasible solutions for the MD-GmTSP in a relatively short amount of time, followed by using a combination of variable neighborhood search (VNS) metaheuristic [39] for improving solution quality (Section 5).
- We explore learning-based methods to solve the MD-mTSP. An architecture consisting of a shared graph neural network and distributed policy networks is used to define a learning policy for MD-GmTSP. Reinforcement learning is used to learn the allocation of agents to vertices to generate feasible solutions for the MD-GmTSP, thus eliminating the need for accurate ground truth (Section 6).
- Finally, we implement all the algorithms on a set of modified instances from the TSPLIB library [40] using CPLEX and present simulation results to corroborate the performance of the proposed approaches (Section 7).

## 2. Literature Review

The traveling salesman problem (TSP) is one of the most well-known optimization problems in the literature [41]. Several methods, ranging from exact techniques (branch and bound/cut/price) [41] to fast heuristics and approximation algorithms [42,43], have been developed to solve the TSP. Heuristics generally aim to trade off optimality for computational efficiency. The mTSP deals with a generalization of the TSP with multiple vehicles. mTSPs are much harder to solve to optimality compared to the single TSP because targets have to be allocated to vehicles in addition to finding a tour for each of the vehicles. Numerous variants and generalizations of the mTSP have been addressed in the literature. For example, mTSP with motion constraints are addressed in [24,44]; fuel constraints are addressed in [45]; capacity and other resource constraints are addressed in [46,47]. Many recent methods have focused on the application of metaheuristics like genetic algorithms (GA) [48,49], simulated annealing (SA) [50], memetic search [51], tabu search [52], swarm optimization [53], and other methods [54,55] to solve the mTSP problem for the single-depot and multi-depot case.

The mTSP problem can be classified into two main types based on its objectives: the MinSum TSP and the MinMax TSP. In MinSum mTSP, the goal is to minimize the sum of the path costs for visiting all targets by the *m* vehicles, while in the MinMax mTSP [56,57,58], the objective is to minimize the maximum among the *m* path costs. While there are several approximation algorithms for the MinSum mTSP [24], there are very few theoretical results for the MinMax mTSP [59,60,61]. Even further, the MinMax mTSP for vehicles with turning radius constraint (Dubins-type vehicle) has received limited or no attention in the literature primarily due to the computational complexity involved in solving the problem.

For the case when all the vehicles are homogeneous without motion constraints, there are several heuristics [62,63,64,65,66] in the literature for solving MinMax mTSPs and related vehicle routing problems. In [62], the authors present four heuristics and found that a linear program (LP)-based heuristic in combination with load balancing and region partitioning ideas performed the best. In [63], the authors present an ant-colony-based metaheuristic to solve the min-max problem with limited computational experiments. A transformation-based method by decoupling the task partitioning problem amongst the vehicles and the routing problems is presented in [65]. In [66], a new heuristic denoted as MD is presented for a min-max vehicle routing problem. Through extensive computational results, the MD algorithm is shown to be the best amongst the considered min-max algorithms in [66].

Recently, MinMax mTSPs with other constraints have also been considered in surveillance applications. In [67], a mixed-integer linear program (MILP) based approach is presented along with market based heuristics for a min-max problem with additional revisit constraints to the targets. In [68], a min-max routing problem is considered where the vehicles are functionally heterogeneous, i.e., there are vehicle-target assignment constraints that specify the sub-set of targets that each vehicle can visit. Both approximation algorithms and heuristics were presented in [68]. In [69], insertion based heuristics are presented for a team of underwater autonomous vehicles with functional heterogeneity. An energy-aware VNS is presented for a vehicle routing problem in [70] where there is a fuel or energy constraint for each of the vehicles. There is also recent work [71] on generalized min-max routing problems where the targets are partitioned into sets, and a vehicle is only required to visit exactly one target from each set.

## 3. Problem Statement

Let $T=\{T_{1},T_{2},\cdots T_{t}\}$ be the set of *t* targets on a 2D plane. Each target is associated with *h* candidate heading angles. For simplicity, lets assume that these heading angles are obtained by sampling the interval [0,2π] uniformly, i.e., $\Phi =\{0,\frac{2\pi }{h},\cdots ,c\frac{2\pi }{h},\cdots ,2\pi \}$, where c≤h. Let $\left\{T_{0}\right\}$ denote the initial target (depot). Let $\left(x_{i},y_{i}\right)$ denote the location of target $T_{i}$, and $\theta_{i}\in \Phi$ denote the arrival angle of a vehicle at target *i*. The angle of arrival and angle of departure of a vehicle from a target is assumed to be the same. To simplify the presentation, we also refer to $\left(x_{i},y_{i}\right)$ as target $T_{i}$. The configuration of the vehicle at a target $T_{i}$ at an angle of $\theta_{i}$ is denoted by $\left(x_{i},y_{i},\theta_{i}\right)$ or simply $\left(T_{i},\theta_{i}\right)$.

Let ν={1,2,⋯m} be the set of *m* homogeneous vehicles with a minimum turning radius ρ. Let $\tau_{k}\subset T$ be the set of targets toured by vehicle *k* visiting each target exactly once, starting and ending at depot $T_{0}$. Let $d_{k}\left(T_{i},\theta_{i},T_{j},\theta_{j}\right)$ denote the shortest Dubins path traversed by vehicle k∈ν, from $\left(T_{i},\theta_{i}\right)$ to $\left(T_{j},\theta_{j}\right)$. Since the vehicles are homogeneous, $d_{k}\left(T_{i},\theta_{i},T_{j},\theta_{j}\right)$ remains the same for all k∈ν. Let the heading angle at the depot for all the vehicles be the same and be given. The objective of MD-GmTSP is to find a tour for each vehicle and the heading angle at each target in *T* such that

- Each target in *T* is visited once by some vehicle at a specified heading angle;
- The tour for each vehicle starts and ends at the depot;
- The length of the longest Dubins tour among the *m* vehicles is minimized.

## 4. Mixed-Integer Linear Programming Formulation

To model the MD-GmTSP as a mixed-integer linear programming problem, we define the related sets, decision variables, and parameters as follows:

### 4.1. Notations

- V:={0,1,2,…,n}, the set of all possible Dubins vehicle configurations. Let *V* be partitioned into mutually exclusive and exhaustive non-empty sub-sets where $V_{0}:=\left\{0\right\}$ corresponds to the configuration at the depot, and $V_{1},\ldots ,V_{t}$ corresponds to the *t* target clusters.
- A:=(i,j):i,j∈V,i≠j, the set of arcs representing the Dubins path between configurations.
- ν:={1,2,⋯,m}, the set of homogeneous (uniform) vehicles that serve these customers.
- $c_{kij}$, the cost of traveling from configuration *i* to configuration *j* for vehicle *k*, where k∈ν, i≠j, $i\in V_{p}$, $j\in V_{r}$, p≠r, p,r∈T.
- $u_{kp}:$, the rank order (visit order) of cluster $V_{p}$ on the tour of vehicle k∈ν, p∈T.
- Q:=t−m+1, the maximum number of clusters that a vehicle can visit.

### 4.2. Decision Variables

Let us define the following binary variables:

$$x_{kij}=\left\{\begin{array}{cc} 1 & \mathrm{if}\;\mathrm{arc}\;\left(i,j\right)\;\mathrm{is}\;\mathrm{included}\;\mathrm{in}\;\mathrm{the}\;\mathrm{tour}\;\mathrm{of}\;\mathrm{vehicle}\;k\in \nu ,i\in V_{p},j\in V_{r},p,r\in T\\ 0 & \mathrm{otherwise} \end{array}\right.$$

$$y_{kpr}=\left\{\begin{array}{cc} 1 & \mathrm{if}\;\mathrm{path}\;\mathrm{exists}\;\mathrm{from}\;\mathrm{target}\;\mathrm{cluster}\;V_{p}\;\mathrm{to}\;\mathrm{cluster}\;V_{r}\;\mathrm{in}\;\mathrm{tour}\;k,p,r\in T,k\in \nu \\ 0 & \mathrm{otherwise} \end{array}\right.$$

### 4.3. Cluster Degree Constraints

For each target cluster $V_{p}$ (excluding $V_{0}$), there can only be a single outgoing arc to any other target cluster belonging to the tour of any of the *m* vehicles. This condition is imposed by the following constraints:

(2) $$\sum_{k\in \nu }\sum_{i\in V_{p}}\sum_{j\in V/V_{p}}x_{kij}=1,\;p\neq 0,p\in T.$$

There can only be a single incoming (entering) arc to a target cluster from any other vertex belonging to other target clusters, excluding $V_{0}$ for a given vehicle. This condition is imposed by the following constraints:

(3) $$\sum_{k\in \nu }\sum_{i\in V/V_{p}}\sum_{j\in V_{p}}x_{kij}=1,\;p\neq 0,p\in T.$$

For each vehicle, there should be a single leaving arc from and a single entering arc to the depot, which are imposed by

(4) $$\sum_{i=1}^{n}x_{k0i}=m,\;k\in \nu .$$

(5) $$\sum_{i=1}^{n}x_{ki0}=m,\;k\in \nu .$$

### 4.4. Cluster Connectivity Constraints

The entering and leaving nodes should be the same for a given vehicle in each cluster, which is satisfied by  

(6) $$\sum_{i\in V/V_{p}}x_{kij}=\sum_{i\in V/V_{p}}x_{kji}\;j\in V_{p},p\in T,k\in \nu .$$

Flows from target cluster $V_{p}$ to target cluster $V_{r}$ for vehicle *k* are defined by $y_{kpr}$. Thus, $y_{kpr}$ should be equal to the sum of $x_{kij}$’s from $V_{p}$ to $V_{r}$. Hence, we write

(7) $$y_{kpr}=\sum_{i\in V_{p}}\sum_{j\in V_{r}}x_{kij},\;p\neq r,\forall p,r\in T,k\in \nu .$$

### 4.5. Sub-Tour Elimination Constraints

The sub-tour elimination constraints and capacity bounding constraints are proposed using the auxiliary variables $u_{kp}$ and *Q* as

(8) $$u_{kp}+\sum_{r=1,r\neq p}y_{rp}\geq 1\;p\neq 0,p\in T,k\in \nu .$$

(9) $$u_{kp}+\left(Q-1\right)y_{kpr}\leq Q,\;p\neq 0,p\in T,k\in \nu .$$

(10) $$u_{kp}-u_{kr}+Qy_{kpr}+\left(Q-2\right)y_{krp}\leq Q-1,\;p\neq r\neq 0,\;p,r\in T,k\in \nu .$$

### 4.6. Objective

The integer linear programming formulation for MD−GmTSP is given by

$$\left(\mathrm{MD}-\mathrm{GmTSP}\right):\mathrm{minimize}\left[\max\left(\sum_{i=0}^{n}\sum_{j=0}^{n}c_{kij}x_{kij}\right)\right],\forall k\in \nu$$

subject to (2)–(10).

### 4.7. Solving the MILP

To obtain a globally optimal solution for a problem expressed as an MILP, it is common to use solvers such as Gurobi or CPLEX. Although these solvers are effective in solving smaller instances of the Euclidean mTSP (for example, an instance with 100 nodes and 3–5 robots takes up to 3 h to solve using a MILP formulation [72]), they struggle to scale to larger instances that involve multiple robots, especially for difficult problems such as the MD-GmTSP. In the following section, we introduce a variable-neighborhood-search-based heuristic that leverages the problem’s structure to generate fast and high-quality solutions.

## 5. Heuristics for the MD-GmTSP

In this section, we present a heuristic based on variable neighborhood search (VNS) to solve the MD-GmTSP. VNS is a metaheuristic proposed by Hansen and Mladenovic [39,73] that employs the idea of systematic exploration of neighborhoods of an initial solution and improves it using local search methods. The neighborhood exploration involves descent to a local minimum in the neighborhood of the incumbent solution or an escape from the valleys containing them to obtain better solutions (global minimum).

The heuristic approach can be divided into two broad phases: (1) construction phase—initial solution and (2) improvement phase—neighborhood search. In the construction phase, a tour construction heuristic is used to generate an initial feasible solution for the given problem. Based on this initial solution, the neighborhood search phase aims to obtain better solutions by making incremental improvements. The tour improvement phase starts by defining a set of neighborhood structures in the solution space. These neighborhood search structures, denoted as $N_{1},N_{2},\cdots ,N_{\kappa_{max}}$, can be chosen either arbitrarily or based on a sequence of neighborhood changes with increasing cardinality. Once the neighborhood search structures are fixed, different neighborhoods are explored in deterministic or stochastic ways, depending on the type of VNS metaheuristic used. If a better solution is found in the κ-th neighborhood, then the search is re-centered around the new incumbent. Otherwise, the search moves to the next neighborhood, κ+1. Depending on the chosen neighborhood structures, different neighborhoods of the solution space are reached. VNS does not follow a predefined trajectory but explores increasingly distant neighborhoods of the current solution. A jump to a new solution is made only if an improvement is achieved, often retaining favorable characteristics of the incumbent solution to obtain promising neighboring solutions.

### 5.1. Initial Solution: Construction Phase

Construction heuristics are commonly used to address vehicle routing problems, and they can be broadly categorized into three classes: insertion heuristics, savings heuristics, or clustering heuristics. In this work, we focus on insertion-based heuristics for the MD-GmTSP, where the algorithm starts with a closed sub-tour comprising a few targets and progressively inserts new targets into the tour until all the targets are visited. Specifically, we explore two types of insertion heuristics for the MD-GmTSP: (1) greedy k-insertion for MD-GmTSP and (2) cheapest k-insertion for MD-GmTSP.

The key differentiator between these algorithms is the order of target selection and the position at which each target is inserted. The heuristics begin with a tour starting at a depot (initial location). A new target is inserted into the tour such that the increase in tour length is minimized. Greedy insertion algorithms, namely Nearest Insertion and Farthest Insertion, select an unvisited target whose distance from any target in the current tour is minimum and maximum, respectively, for insertion. In contrast, the cheapest-insertion algorithm selects an unvisited target whose insertion causes the lowest increase in tour length. The main steps involved in these construction heuristics for the MD-GmTSP are discussed below.

Consider a set of *t* targets and their associated heading angles, $V=\{\left(T_{1},\theta_{1}\right),\cdots ,\left(T_{i},\theta_{i}\right),$$\cdots ,(T_{t},\theta_{t})\}$ to be visited by a *m* Dubins vehicles, and let $\left(T_{0},\theta_{0}\right)$ correspond to the depot target. To simplify the presentation, we also refer to any target configuration $\left(T_{i},\theta_{i}\right)$ as a target. Let $\tau_{v}=\{\left(T_{0},\theta_{0}\right),\cdots ,\left(T_{i},\theta_{i}\right),$$\cdots ,(T_{0},\theta_{0})\}$, i∈[1,p] denote a Dubins tour for vehicle v∈ν, through p(≤t) cities, starting and ending at the depot. Let $d_{v}\left(T_{i},T_{i+1}\right)$ denote the length of the shortest Dubins path from $\left(T_{i},\theta_{i}\right)$ to $\left(T_{i+1},\theta_{i+1}\right)$. Consider a target $\left(T_{j},\theta_{j}\right)$ from the set of unvisited targets. The increase in tour length by inserting the $T_{j}$ between targets $T_{i}$ and $T_{i+1}$ in tour $\tau_{v}$, without changing the associated heading angles, would be $\Delta D_{v}\left(T_{j}\right)=d_{v}\left(T_{i},T_{j}\right)+d_{v}\left(T_{j},T_{i+1}\right)-d_{v}\left(T_{i},T_{i+1}\right)$. We wish to insert the new unvisited target $T_{j}$ in a tour $\tau_{v}$ at a position such that the increase in length of tour *v* ($\Delta D_{v}\left(T_{j}\right)$) results in a minimum increase in length of the largest of the *m* tours. One way of achieving this is by greedily inserting the new target $T_{j}$ in the smallest of the *m* tours without affecting the rest of the tour(s).

A relaxed version of the above approach is to allow modifications to the heading angles of k(≥0) targets adjacent to the newly inserted target such that the increase in tour length is minimum. Given a Dubins tour $\tau_{v}=\left\{\left(T_{0},\theta_{0}\right),\cdots ,\left(T_{p},\theta_{p}\right),\cdots ,\left(T_{0},\theta_{0}\right)\right\}$, let $D_{v}\left(T_{i-k},T_{(i+1)+k}\right)$ be the length of the shortest possible Dubins sub-tour through targets $T_{i-k},\ldots ,T_{i},T_{i+1},\ldots ,T_{(i+1)+k}$ with predefined headings $\theta_{i-k},\cdots ,\theta_{(i+1)+k}$. Then, $\Delta D_{v}\left(T_{j}\right)=D\left(T_{i-k},T_{j}\right)+D\left(T_{j},T_{(i+1_{+}k}\right)-D\left(T_{i-k},T_{(i+1)+k}\right)$ describes the increase in the tour length of vehicle *v* by inserting target $T_{j}$. This version allows for the heading angles of 2k targets between $T_{i-k}$ and $T_{(i+1)+k}$ to be optimized when the target $T_{j}$ in inserted into the tour. Algorithm 1 presents a formal description of the k−greedy MD-GmTSP tour construction heuristic.
**Algorithm 1** Greedy k-insertion for MD-GmTSP.*Initialization*: Start with *m* initial Dubins tours $\tau_{v},\forall v\in \left[1,m\right]$ starting and ending at the depot $\left(T_{0},\theta_{0}\right)$.*Repeat*: While the set of unvisited targets $N_{u}$ is non-empty1.Identify the tour with the shortest Dubins tour length $\tau_{\nu_{min}}\leftarrow \min\left(D_{v}\right),\forall v\in \left[1,m\right]$2.Choose a target $T_{j}$ from the list of unvisited targets $N_{u}$ according to one of the following greedy strategy.
(a)Random: Choose a random target $T_{j}\in N_{u}$(b)Nearest: Find a target $T_{j}\in N_{u}$ such that the Dubins distance $d(T_{j},T_{j^{\prime }})$ is minimum $\forall T_{j^{\prime }}\in \tau_{\nu_{min}}$(c)Farthest: Find a target $T_{j}\in N_{u}$ such that the Dubins distance $d(T_{j},T_{j^{\prime }})$ is maximum $\forall T_{j^{\prime }}\in \tau_{\nu_{min}}$
3.Determine a position *i* in the tour $\tau_{\nu_{min}}$ such that the increase in tour length $\Delta D_{\nu_{\min}}\left(T_{j}\right)=D\left(T_{i-k},T_{j}\right)+D\left(T_{j},T_{(i+1_{+}k}\right)-D\left(T_{i-k},T_{(i+1)+k}\right)$ is minimum; insert $T_{j}$ in $\tau_{\nu_{min}}$.4.Remove $T_{j}$ from the set of unvisited targets $N_{u}$

In the cheapest k-insertion method (Algorithm 2), during each iteration, the algorithm finds the most promising target (from the set of unvisited targets) and the position for insertion in the current tour simultaneously. In this algorithm, we construct new tours by inserting new targets at positions that lead to the smallest increase in tour lengths.
**Algorithm 2** Cheapest k-insertion for MD-GmTSP*Initialization*: Start with *m* initial Dubins tours $\tau_{v},\forall v\in \left[1,m\right]$ starting and ending at the depot $\left(T_{0},\theta_{0}\right)$.*Repeat*: While the set of unvisited targets $N_{u}$ is non-empty1.Identify the tour with the shortest Dubins tour length $\tau_{\nu_{min}}\leftarrow \min\left(D_{v}\right),\forall v\in \left[1,m\right]$2.Identify the target $T_{j}$ from the list of unvisited targets $N_{u}$, and a position *i* in the tour $\tau_{\nu_{min}}$ such that the increase in tour length $\Delta D_{\nu_{min}}\left(T_{j}\right)=D\left(T_{i-k},T_{j}\right)+D\left(T_{j},T_{(i+1_{+}k}\right)-D\left(T_{i-k},T_{(i+1)+k}\right)$ is minimum; insert $T_{j}$ in $\tau_{\nu_{min}}$.3.Remove $T_{j}$ from the set of unvisited targets $N_{u}$

### 5.2. Neighborhood Search: Improvement Phase

In the improvement phase, we use VNS-based local search heuristics to enhance the incumbent solution from the construction phase. These heuristics modify the current solution by performing a sequence of operations to produce a new feasible solution that improves the objective function. The algorithm evaluates the effect of modifying the current solution systematically and replaces it with a new solution if it is better. The algorithm may also be allowed to make changes that lead to poorer solutions with the hope of finding a better solution later.

Let *I* denote a problem instance of the MD-GmTSP. Let *X* represent the set of feasible solutions for this instance. For any x∈X, let f(x) denote a function that calculates the objective cost, which, in the case of the MD-GmTSP, corresponds to the Dubins tour length of the largest tour within *x*. It is important to note that the solution set *X* is finite but the size of the set can exponentially increase with the size (number of targets) of an instance of MD-GmTSP. Our goal is to find a solution $x^{\prime }$ such that $f\left(x^{\prime }\right)\leq f\left(x\right),\forall x\in X$. To facilitate this search, we define a neighborhood N(x)⊂X for each solution x∈X, where *N* is a function that maps from a solution *x* to a set of solutions in its neighborhood. A solution x∗ is defined to be locally optimal or is said to be a local optimum with respect to a neighborhood *N* if $f\left(x*\right)\leq f\left(x^{\prime }\right),\forall x^{\prime }\in N\left(x*\right)$. Improvement heuristics aim to find a locally optimal solution before the termination condition is met. In this paper, we investigate three improvement heuristics based on VNS for solving the MD-GmTSP, namely (1) basic variable neighborhood search (BVNS), (2) variable neighborhood descent (VND), and (3) general variable neighborhood search (GVNS).

#### 5.2.1. Basic Variable Neighborhood Search (BVNS)

BVNS [39] uses a stochastic approach to reach the global minimum. In this approach, the neighborhood search is continued in a random direction, away from the initial solution. The steps of BVNS are explained in Algorithm 3.
**Algorithm 3** Steps of the basic VNS by Hansen and Mladenovic  [73]Initialization: Choose a set of neighborhood structures denoted by $N_{k}\left(\kappa =1,\ldots ,\kappa_{max}\right)$ for the search process; find an initial solution *x*; select a termination/stopping condition and repeat the following steps until the termination condition is met:1.Set κ←1.2.Repeat the following steps until $\kappa =\kappa_{\max}$:
(a)*Shaking*: Generate a feasible solution $x^{\prime }$ at random from the κth neighborhood of $x(x^{\prime }\in N_{k}\left(x\right))$;(b)*Local search*: Apply a local search method with $x^{\prime }$ as an initial solution; denote the obtained local optimum as $x^{\prime \prime }$;(c)*Move or not*: If $x^{\prime \prime }$ is better than the incumbent *x*, set $x\leftarrow x^{\prime \prime }$ and continue the search process with $N_{1}\left(\kappa \leftarrow 1\right)$; otherwise, set κ←κ+1;


##### Neighborhood Structures

In this work, we use k−exchange operators to explore neighborhoods of MD-GmTSP using BVNS. A solution $\chi^{\prime }$ is considered to be in the k−exchange neighborhood of solution χ if there is exactly one vehicle $V_{i}$ such that:

(1) $\chi^{\prime }$ and χ differ only in the order of target visits in the tour of vehicle $V_{i}$.
(2) The tour for vehicle $V_{i}$ in $\chi^{\prime }$ differs from the tour of vehicle $V_{i}$ in χ by at most *k* edges.

##### Shake

**Figure 3:**
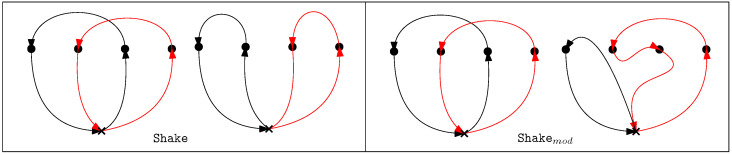
Different Shake neighborhoods. The black and the red paths indicate the tours of the vehicles involved in the shake process.

‘Shaking’ is the randomization part of the BVNS heuristic. Shaking perturbs the initial solution while ensuring that the new neighborhood still retains certain aspects of the initial incumbent. In this step, a random feasible solution $x^{\prime }$ in the current neighborhood of *x* is found. This random selection enables the algorithm to avoid stopping at the local optima in the local search procedure. In this work, we investigate two different Shake neighborhoods: Shake and Shake${}_{mod}$. Shake refers to picking two random targets $T_{j}$, $T_{j}^{{}^{\prime }}$ from tours of two random vehicles *v* and $v^{\prime }$, respectively, and swapping the targets between tours. The position of insertion for the targets is also selected at random. For the MD-GmTSP, in addition to Shake, a modified shake Shake${}_{mod}$ is also evaluated. In Shake${}_{mod}$, a random target $T_{j}$ is selected from a random tour $\tau_{v}$, and is transferred to another tour $\tau_{v}^{{}^{\prime }}$ chosen at random. The insertion of the targets into the tours can be random or greedy (position leading to a minimal increase in tour length). The different shake neighborhoods are illustrated in Figure 3.

##### Local Search

After obtaining the solution $x^{\prime }$ from the Shake procedure, we proceed to enhance it through local search. During this step, all feasible neighbors of $x^{\prime }$ within the current neighborhood are explored to identify a potential solution $x^{\prime \prime }$ that has a lower cost. It is worth noting that there is a possibility for the current neighborhood of $x^{\prime }$ to be empty. In that case, we set $x^{\prime \prime }$ to be the same as $x^{\prime }$, and the search continues in the next neighborhood. Exploration of neighborhoods can be of two types: (a) steepest descent and (b) first descent. The steepest descent and first descent heuristics are detailed in Algorithms 4 and 5, respectively.

For a strong NP-hard problem like the MD-GmTSP, the steepest descent heuristic is a highly time-consuming process. In this work, a first improvement version of the 2-opt and 3-opt algorithms explores the different neighborhood structures of the MD-GmTSP.
**Algorithm 4** SteepestDescent Heuristic by Hansen and Mladenovic [73]Initialization: Find an initial solution *x*; Choose the neighborhood structures N(x), and the objective function f(x) that calculates the length of the longest tour in *x*,Repeat:1.Find $x^{\prime }=$*arg*$\min_{x\in N(x)}f\left(x\right)$2.If $f\left(x^{\prime }\right)<f\left(x\right)$ set $x^{\prime }\leftarrow x^{\prime \prime }$ and iterate; otherwise, stop

**Algorithm 5** FirstImprovement heuristic by Hansen and Mladenovic [73]
Initialization: Find an initial solution *x*; Choose the neighborhood structures N(x), and the objective function f(x) that calculates the length of the longest tour in *x*,
Repeat:1.Find first solution $x^{\prime }\in N\left(x\right)$2.If $f\left(x^{\prime }\right)>f\left(x\right)$ find next solution $x^{\prime \prime }\in N\left(x\right)$; set $x^{\prime }\leftarrow x^{\prime \prime }$ and iterate (2); otherwise set $x\leftarrow x^{\prime }$ and iterate (1);3.If all solutions of N(x) have been considered, stop.


##### 2-opt

**Figure 4:**
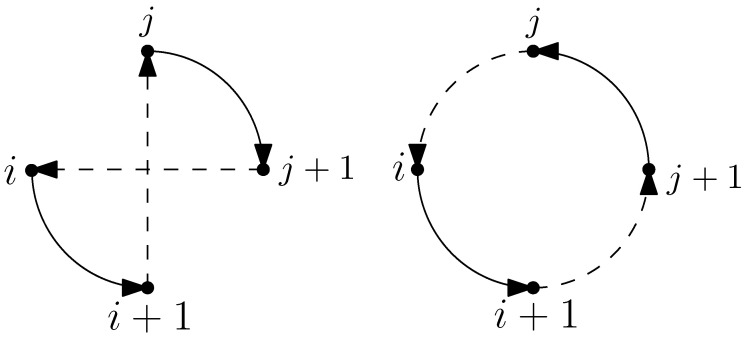
A sample 2-opt move for a given tour.

Given a solution, the 2-opt algorithm, introduced by Croes in 1958 [74], is a local search heuristic that explores all possible 2-Exchange neighborhoods of the solution. Its objective is to gradually enhance a feasible tour by iteratively swapping two edges in the current tour with two new edges, aiming to reach a local optimum where no further improvements are possible. During each improving step, the 2-opt algorithm selects two distinct edges that connect $\left(i,i^{\prime }\right)$ and $\left(j,j^{\prime }\right)$ from the tour. It then replaces these edges with the edges connecting (i,j) and $\left(i^{\prime },j^{\prime }\right)$, provided that this change reduces the tour length. This process continues until a termination condition is encountered (See Figure 4).

##### 3-opt

**Figure 5:**
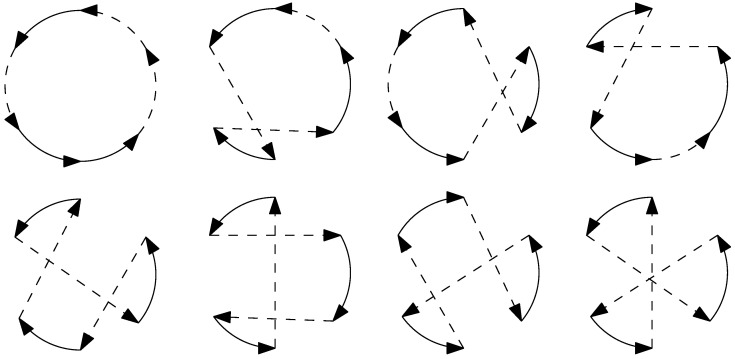
All possible combinations of 3-Opt moves for a given tour.

The 3-opt algorithm [75] works similarly to the 2-opt but by reconnecting three edges instead of two, as seen in Figure 5. There are seven different ways of reconnecting the edges. Each new tour formed by reconnecting the edges is analyzed to find the optimum one. This process repeats until all possible three-edge combinations in the network are checked for improvement. A 3-optimal tour is also a 2-optimal one. A 3-opt exchange yields better solutions, but it is much slower ($O(n^{3})$ complexity) compared to the 2-opt ($O(n^{2})$ complexity).

On completion of the local search, the cost of $x^{\prime \prime }$ is compared to the cost of *x*. If the cost of $x^{\prime \prime }$ is less than the cost of *x*, then $x^{\prime \prime }$ is set to be *x*. The current neighborhood of $x^{\prime \prime }$ is set as the first neighborhood (κ←1), and the algorithm continues from the shaking step. On the contrary, if the cost of $x^{\prime \prime }\geq x$, then the solution $x^{\prime \prime }$ is discarded and search continues in the next neighborhood κ+1. The algorithm terminates when there are no new neighborhoods to be explored. The steps of neighborhood change are detailed in Algorithm 6.
**Algorithm 6** Steps for neighborhood change by Hansen and Mladenovic [73]**function** neighborhoodChange(*x*, $x^{\prime }$*k*)    **if** $f\left(x^{\prime }\right)<f\left(x\right)$ **then**        $x\leftarrow x^{\prime }$; k←1                             ▹ Make a move    **else**        k←k+1                            ▹ Next neighborhood

#### 5.2.2. Variable Neighborhood Descent (VND)

The main difference between the BVNS and the VND approach is that in the VND, the neighborhood changes are deterministic. Algorithm 7 explains the VND metaheuristic in detail.

Depending on the problem, multiple local search methods can be nested to optimize the VND search. The objective of the local search heuristics is to find the local minimum among all the $l_{max}$ neighborhoods. As a result, the likelihood of reaching a global minimum is higher when using VND with a larger $l_{max}$ than when using a single-neighborhood structure.
**Algorithm 7** Steps of the basic VND by Hansen and Mladenovic  [73]**Initialization:** Choose a set of neighborhood structures denoted by $N_{l}\left(l=1,\ldots ,l_{max}\right)$ that will be used in the descent process; find an initial solution *x*; select a termination/stopping condition and repeat the following steps until the termination condition is met:1.Set l←1.2.*Repeat* the following steps until $l=l_{\max}$:
(a)*Exploration of neighborhood*: Find the best neighbor $x^{\prime }$ of *x* ($x^{\prime }\in N_{l}\left(x\right)$);(b)*Move or not*: If $x^{\prime \prime }$ is better than the incumbent *x*, set $x\leftarrow x^{\prime \prime }$ and continue the search process with $N_{1}\left(l\leftarrow 1\right)$; otherwise set l←l+1;


##### Exploration of Neighborhoods for MD-GmTSP

We use a combination of four different neighborhood structures to explore the neighborhoods for MD-GmTSP. An *x*-point move is nested with a *k*-opt search (2-opt or 3-opt) to explore distant neighborhoods during local search coupled with greedy and random insertion techniques. The neighborhood structures are discussed in detail below.

- One-point move: Given a solution *x*, a one-point move transfers a target $T_{j}$ from tour $\tau_{v}$ to a new feasible position in another tour $\tau_{v^{\prime }}\;(\neq$$\tau_{v})$. The target to be moved is chosen from the tour having the largest tour length and is relocated to a tour having the smallest tour length. The computational complexity of these local search operators is $O(n^{2})$ for a given solution $x^{\prime }$.
- Two-point move: A two-point move swaps a pair of nodes rather than transferring a node between tours as in a one-point move. A target $T_{j}$ belonging to the tour $\tau_{v}$ having the largest tour length is swapped with a target $T_{j^{\prime }}$ belonging to another tour $\tau_{v^{\prime }}\;(\neq$$\tau_{v})$. After performing two-point moves from the solution $x^{\prime }$, the best solution $x^{\prime \prime }$ is returned depending on the search strategy employed (FirstImprovement or SteepestDescent).

#### 5.2.3. General Variable Neighborhood Search (GVNS)

The general variable neighborhood search (GVNS) [73] merges the techniques used in both VND (deterministic descent to a local optimum) and BVNS (stochastic search) to reach increasingly distant neighborhoods in the search space. In GVNS, a sequential VND search ($VND_{seq}$) improves the initial solution as compared to a local search heuristic (as in the case of BVNS). Algorithm 8 details the steps of GVNS as proposed by Hansen and Mladenovic.
**Algorithm 8** Steps of the GVNS by Hansen and Mladenovic [73]*Initialization*: Choose the set of neighborhood structures denoted by $N_{\kappa }\left(\kappa =1,\cdots ,\kappa_{max}\right)$ that will be used in the shaking phase and the set of neighborhood structures $N_{l}\left(l=1,\cdots ,l_{max}\right)$ that will be used in the local search process; find an initial solution *x*; choose a termination/stopping condition.*Repeat* the following steps until the termination condition is met:1.Set κ←1.2.*Repeat* the following steps until $\kappa =\kappa_{\max}$:
(a)*Shaking*: Generate a feasible solution $x^{\prime }$ at random from the κth neighborhood of $x(x^{\prime }\in N_{k}\left(x\right))$;(b)*Local search by VND*:
Set l←1*Repeat* the following steps until $l=l_{max}$
-*Exploration of neighborhood.* Find the best neighbor $x^{\prime \prime }$ of $x^{\prime }$ in $N_{l}\left(x^{\prime }\right)$-*Move or not.* If $f\left(x^{\prime \prime }\right)<f\left(x^{\prime }\right)$, set $x^{\prime }\leftarrow x^{\prime \prime }$ and l←1; otherwise set l←l+1
(c)*Move or not*: If the local optimum $x^{\prime \prime }$ is better than the incumbent, move there $\left(x\leftarrow x^{\prime \prime }\right)$ and continue the search with $N_{1}\left(\kappa \leftarrow 1\right)$; otherwise set κ←κ+1;

##### Neighborhood Search Structures for MD-GmTSP

**Figure 6:**
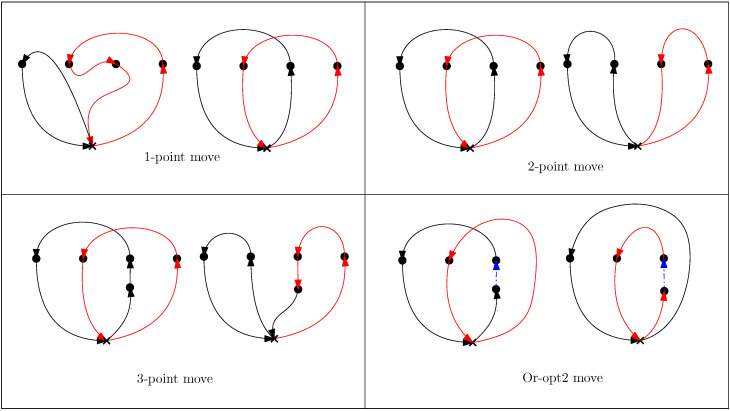
Local search operators. The black and the red paths denote the tours of the vehicles involved in each move. The blue arrow in the Or-opt2 move shows the string of two adjacent nodes that is transferred between tours.

To ensure the effectiveness of GVNS, it is crucial to employ suitable local search strategies during the execution of **Local Search by VND** ($VND_{seq}$). The performance of $VND_{seq}$ is influenced by the choice of neighborhood structures and the order in which these neighborhoods are explored. A combination of x−point and k−edge move neighborhoods are used in this paper to solve the MD-GmTSP. The details of these neighborhoods are provided below and illustrated in Figure 6.

1. **One-point move**: In GVNS, we use the same One-point move operator as in BVNS.
2. **Two-point move**: In GVNS, we use the same Two-point move operator as in BVNS.
3. **Or-opt2 move**: An or-opt2 move selects a string of two adjacent nodes belonging to the tour having the maximum length and transfers it into a new tour. After performing the or-opt2 move for all strings of nodes $x^{\prime }$, the best solution $x^{\prime \prime }$ is returned by the operator depending on the search strategy employed. The *or-opt*2 operator generates $x^{\prime \prime }\in N_{2}\left(x^{\prime }\right)$ as compared to generic or-opt*k* move operator generates $x^{\prime \prime }\in N_{k}\left(x^{\prime }\right)$. For our use case, no improvement was observed for k>2, which can be attributed to the complexity in the MD-GmTSP problem.
4. **Three-point move**: The three-point move operation involves selecting a pair of adjacent nodes from the tour with the maximum length and exchanging them with a node from another tour. By repeatedly applying three-point moves starting from the initial solution $x^{\prime }$, the operator generates a new solution $x^{\prime \prime }$. Depending on the chosen search strategy, the best solution $x^{\prime \prime }\in N_{3}\left(x^{\prime }\right)$ is returned by the operator.
5. **2-opt move**: We use a 2-opt move operator to improve the resulting tours $x^{\prime \prime }$ obtained from the rest of the operators by performing intra-tour local optimization.

##### Local Search by VND for MD-GmTSP

A nested local search strategy is employed to solve the MD-GmTSP using GVNS. The $VND_{seq}$ as specified in Algorithm 9 explores the neighbors of a given solution according to a particular sequence. This sequence is determined based on the non-decreasing order of neighborhood size. Based on the local search in each neighborhood, if the obtained solution ($x^{\prime \prime }$) is better than the current best ($x^{\prime }$), the 2-opt search is carried out for possible intra-tour improvements. The resulting solution replaces the current best ($x^{\prime }\leftarrow x^{\prime \prime }$), and the search restarts at the first neighborhood. Otherwise, the search continues with a larger neighborhood search operator.
**Algorithm 9** 
$VND_{seq}$
1:**procedure**$VND_{seq}$($x^{\prime }$, $l_{max}$)2:    l←13:    **while** $l<l_{max}$ **do**4:        **if** l=1 **then**5:           $x^{\prime \prime }\leftarrow$onepoint$\left(x^{\prime }\right)$6:        **if** l=2 **then**7:           $x^{\prime \prime }\leftarrow$twopoint$\left(x^{\prime }\right)$8:        **if** l=3 **then**9:           $x^{\prime \prime }\leftarrow$or_opt2$\left(x^{\prime }\right)$10:        **if** l=4 **then**11:           $x^{\prime \prime }\leftarrow$threepoint$\left(x^{\prime }\right)$12:        **if** $f\left(x^{\prime \prime }\right)<f\left(x^{\prime }\right)$ **then**13:           $x^{\prime \prime }\leftarrow 2\_opt\left(x^{\prime \prime }\right)$14:           $x^{\prime }\leftarrow x^{\prime \prime }$; l←115:        **else**16:           l←l+117:    **return** $x^{\prime }$

Using heuristics to solve NP-hard problems provides a distinct advantage over traditional solvers as it can produce feasible solutions quickly. The quality of the solution can be further improved by employing improvement heuristics, but this often comes at the cost of computational power and time. Heuristics are rule-based approaches that prioritize improving computational efficiency over finding the optimal solution. Recently, there has been significant interest in learning-based approaches from the combinatorial optimization research community. These approaches utilize models that can solve large problem instances in real-time. The concept behind this approach is that heuristics can be viewed as decision-making policies which can be parameterized using neural networks. The policies can then be trained to solve different combinatorial optimization problems. In the next section, we introduce a reinforcement learning method to develop policies for solving the MD-GmTSP. This method holds great promise for achieving high-quality solutions in a shorter amount of time than traditional heuristics.

## 6. Learning-Based Approach for the MD-GmTSP

Several machine learning techniques, including neural networks [76], pointer networks [77], attention networks [78,79] and reinforcement learning [80,81] have been explored to solve the TSP problem. Wouter et al. [79] extended a single-agent TSP model to learn strong heuristics for the vehicle routing problem (VRP) and its variants. However, supervised learning, which is successful for most machine learning problems, is not applicable for combinatorial optimization problems due to the unavailability of optimal solutions for large instances, especially for co-operative combinatorial optimization problems like multiple agent TSP, asymmetric mTSP, and multi-agent task assignment and scheduling problems. These problems pose several challenges, such as an explosion of the search space with the increase in the number of agents and cities, a lack of data with ground truth solutions, and difficulty in developing an architecture that can capture interactive behaviors among agents. Reinforcement learning (RL) is a commonly explored paradigm to solve problems with the above difficulties, whose solution quality can be verified and provided as a reward to a learning algorithm. Recent studies [82,83] have investigated using RL-based approaches to optimize the Euclidean version of MinMax TSP, but no significant study has been conducted for the Dubins MinMax TSP. The main advantage of using the RL-based method is that the majority of computational time and resources are spent on training. Once a trained model is available, a feasible solution can be inferred almost in real-time.

In this paper, we utilize the RL framework developed by Hu et al. [82] and expand its application to the Dubins MTSP. The model consists of a shared graph neural network and distributed policy networks that collectively learn a universal policy representation for generating nearly optimal solutions for the Dubins MTSP. To address the challenges of optimization, the extensive search space of the problem is effectively partitioned into two tasks: assigning cities to agents with candidate heading angles and performing small-scale Dubins TSP planning for each agent. As proposed by Hu et al. [82], an S-sample batch reinforcement learning technique is used to address the lack of training data, reducing the gradient approximation variance and significantly enhancing the convergence speed and performance of the RL model.

### 6.1. Framework Architecture

In this work, the graph isomorphism network (GIN) [84] policy is used to summarize the state of the instance graph and assigns nodes to each agent for visitation, effectively transforming the MD-GmTSP into a single-agent DTSP problem. This approach facilitates the use of algorithms [85,86], learning-based methods [77,79,80], and solvers [87,88,89] to quickly obtain near-optimal solutions. Additionally, we utilize a group of distributed policy networks to address the node-to-agent assignment problem.

#### 6.1.1. Graph Embedding

For each node v∈V, the graph embedding network GIN [84] computes a *p*-dimensional feature embedding $f_{v}$ by information sharing from the neighboring connected nodes according to the graph structure. GIN uses the following update process to parameterize the graph neural network:

(11) $$f_{v}^{\left(k\right)}=MLP^{\left(k\right)}\left\{\left(1+\epsilon^{\left(k\right)}\right).h_{v}^{\left(k-1\right)}+\sum_{u\in N(v)}^{}h_{u}^{\left(k-1\right)}\right\}$$

where $f_{v}^{k}$ is the feature vector of node v∈V at the k−th iteration/layer, $N_{v}$ denotes all the neighbors of node *v*, *u* is neighboring node of *v* (i.e., $u\in N_{v}$), MLP represents a multi-layer perceptron, and ϵ is a learnable parameter.

#### 6.1.2. Distributed Policy Networks

We propose a two-stage distributed policy network-based approach for designing a graph neural network with the attention mechanism [90]. In the first stage, each agent autonomously generates its *agent embedding* by leveraging both the global information and the node embeddings present in the graph. In the second stage, each node selects an agent to associate with itself based on its own embedding and all the prior agent embeddings.

##### Calculation of Agent Embedding

Graph embeddings, denoted as $g_{f}$, are computed by max pooling from the set of node features $f=\{f_{1},f_{2},\ldots f_{n}\}$, i.e.,

$$g_{f}=\max\left\{f_{1},f_{2},\cdots ,f_{n}\right\}.$$

These graph embeddings are then used as inputs to construct an agent embedding, where $f_{i}\in R^{p}$, *n* is the number of nodes, *p* is the dimension of node embedding, and $g_{f}\in R^{p}$. To construct the agent embedding, an attention mechanism is employed to generate attention coefficients that signify the significance of a particular node’s feature in creating its embedding.

- *Graph Context embedding:* A graph context embedding is used to ensure that every city, except the depot, is visited by only one agent and the depot is visited by all agents. By setting the depot as the first node in the graph ($f_{depot}=f_{1}$), we concatenate the depot features with the global embedding to create the graph context embedding, represented as $f_{c}\in {I\;R}^{2p}$. The concatenation operation is denoted by [.;.] and is shown in Equation (12):
  (12) $$f_{c}=\left[g_{f};f_{1}\right].$$
- *Attention Mechanism:* The attention mechanism [90] is used to convey the importance of a node to an agent *a*. The node feature set obtains the keys and values, and the graph context embedding computes the query for agent *a*, which is standard for all agents.
  $$q_{a}=\theta_{ag}^{d^{k}\times 2p}f_{c}^{2p},$$
  $$k_{ai}=\theta_{ak}^{d^{k}\times p}f_{i}^{p},i=\left\{2,3,\ldots ,n\right\},$$
  (13) $$v_{ai}=\theta_{av}^{d_{v}\times p}f_{i}^{p},i=\left\{2,3,\ldots ,n\right\},$$
  where $d_{k}$ and $d_{v}$ are the dimension of key and values. A *SoftMax* is used to compute the attention weights $w_{ai}\in \left[0,1\right]$, which the agent puts on node *i*, using:
  (14) $$w_{ai}=\frac{e^{u_{ai}}}{\sum_{j}e^{u_{aj}}},i=2,3,\ldots ,n,j=2,3,\ldots ,n,$$
  where $u_{ai}$ is the compatibility of the query to be associated with the agent and all its nodes given by
  (15) $$u_{ai}=\frac{q_{a}^{T}k_{ai}}{\sqrt{d_{k}}},i=2,3,\ldots ,n.$$
- *Agent embedding:* From the attention weights, we construct the agent embedding using
  (16) $$h_{a}=\sum_{j}w_{aj}v_{aj}.$$

In this section, we outline the process for determining the policy that assigns an agent to a specific node. To evaluate the importance of each agent to the node, we utilize the following set of equations. These equations are applied to each agent individually as follows:

$$k_{ai}^{{}^{\prime }}=\theta_{ak^{{}^{\prime }}}^{d_{k}^{{}^{\prime }}\times p}f_{i}^{p},i=2,3,\ldots ,n,$$

$$q_{a}^{{}^{\prime }}=\theta_{aq^{{}^{\prime }}}^{d_{k}^{{}^{\prime }}\times d_{v}}h_{a}^{d_{v}},$$

(17) $$u_{ai}^{{}^{\prime }}=\frac{q_{a}^{T}k_{ai}^{{}^{\prime }}}{\sqrt{d_{k}^{{}^{\prime }}}},$$

(18) $$imp_{ai}=C\tanh\left(u_{ai}^{{}^{\prime }}\right),i=2,3,\ldots ,n.$$

Here, $d_{k}^{{}^{\prime }}$ is the dimension of new keys; $\theta_{ak^{{}^{\prime }}}$ and $\theta_{aq^{{}^{\prime }}}$ are parameters of neural networks to project the embeddings back to $d_{k}^{{}^{\prime }}$ dimensions. The importance $imp_{ai}$ is computed by obtaining $u_{ai^{{}^{\prime }}}$ for a new round of attention and clipping the result within [−10,10] using tanh [80].

Since each agent is limited to visiting only one city, the agent’s importance factor determines which city it visits. A *SoftMax* function evaluates the probability of an agents likelihood to visit a node:

(19) $$p_{ai}=\frac{e^{imp_{ai}}}{\sum_{a}e^{imp_{ai}}}$$

where $p_{ai}$ is the probability of agent *a* visiting node *i*; $imp_{ai}$ is the importance of node *i* for agent *a*; a∈1,2,…,m; and i∈1,2,…,n. The *SoftMax* function is used for creating a probabilistic policy that can be optimized using gradient-based techniques. In the context of a policy network, the parameters of the *SoftMax* function are typically learned from data that reflects the characteristics of the agents being modeled. As such, the policy network may need to be retrained when those characteristics change, such as when different turning radius constraints or a different number of agents are introduced.

##### S-Samples Batch Reinforcement Learning

The parameter θ for our model is estimated by maximizing the expected reward of the policy, i.e.,

$$\theta^{*}=\arg\max_{\theta }L_{R}\left(\theta \right).$$

(20) $$\begin{array}{cc} L_{R}\left(\theta \right) & =E_{(G,m,n)∼D}E_{\lambda ∼\pi (\theta )}R\left(\lambda \right)\\ \; & =E_{(G,m,n)∼D}\sum_{\lambda }\pi_{\theta }\left(\lambda |G,m,n\right)R\left(\lambda \right) \end{array}$$

where *D* is the training set, λ is an assignment of cities representing which agent visits it, R(λ) is the reward of the assignment, and λ, $\pi_{\theta }$ is the distribution of the assignments over θ, i.e., $\pi_{\theta }\left(\lambda \right)=\prod_{i\in \{1,\ldots ,n\}}p_{ai}$. However, the inner sum over all assignments in Equation (20) is intractable to compute. REINFORCE [91] is used as the estimator for the gradient of the expected reward. An S-sample batch approximation decreases variances in the gradient estimate and speeds up convergence.

(21) $$L_{R}\left(\theta \right)\approx E_{(G,m,n)∼D}\sum_{s=1}^{S}\pi_{\theta }\left(\lambda_{s}|G,m,n\right)R\left(\lambda_{s}\right).$$

The variance during training is decreased by introducing an advantage function $A(\lambda_{s})$:

(22) $$A\left(\lambda_{s}\right)\approx R\left(\lambda_{s}\right)-\frac{1}{S}\sum_{z=1}^{S}R\left(\lambda_{z}\right).$$

This results in a new optimization function:

(23) $$\theta^{*}=\arg\max_{\theta }L_{R}\left(\theta \right)\approx \arg\max_{\theta }E_{D}\sum_{s=1}^{S}\pi_{\theta }\left(\lambda_{s}\right)A\left(\lambda_{s}\right),$$

(24) $$\nabla_{\theta }L_{R}\left(\theta \right)\approx E_{D}\sum_{s=1}^{S}\left(\sum_{i=1}^{n}\nabla_{\theta }\log p_{ai}\right)A\left(\lambda_{s}\right).$$

The pyDubins [92] code computes the rewards for every assignment, which calculates the DTSP tour lengths and returns the negative of the maximum tour length over all agents as the reward for the assignment.

## 7. Computational Results

Algorithms were implemented using an Intel NUC Kit (NUC8i7HVK) equipped with an Intel Core i7 processor and 32 GB of RAM. The effectiveness of the proposed methods was evaluated by solving instances from the TSPLIB library [40], which contains sample instances for the TSP and related problems with varying sizes ranging from 16 to 176 cities. To adapt the TSPLIB instances for the MD-GmTSP, each city was associated with a set of h=8 candidate heading angles. The minimum turning radius (ρ) was calculated for each instance based on the layout of the cities. Specifically, ρ was computed as follows:

$$\rho =\lceil 0.1\times \max_{i,j\in I}\left(\sqrt{{\left(y_{j}-y_{i}\right)}^{2}+{\left(x_{j}-x_{i}\right)}^{2}}\right)\rceil$$

where $\left(x_{i},y_{i}\right)$ and $\left(x_{j},y_{j}\right)$ are the 2D coordinates of city *i* and city *j*, respectively, for all i,j∈I.

### 7.1. MILP Results

Each instance was solved using the MILP formulation for MD-GmTSP described in Section 3. The CPLEX 12.10 [87] solver was used with a computational time limit of 10,800 s (3 h) to generate optimal results. Table 1 presents the results of the MD-GmTSP for m=3 homogeneous vehicles and h=8 heading angle discretizations. CPLEX was able to generate feasible solutions within the time limit only for instances with fewer than 52 cities and did not find optimal solutions for any of the instances. The optimality gap for the best result, obtained on the Ulysses-22 instance with 22 cities, was 14.09%.

### 7.2. VNS-Based Heuristics Results

The algorithms discussed in Section 5, including the construction heuristics and the variable neighborhood search (VNS)-based improvement heuristics, were implemented in Python3 and tested on the same set of TSPLIB instances for m=3 vehicles and h=8 (discrete heading angles).

#### 7.2.1. Quality of Initial Feasible Solutions

Table 2 presents a comparison of the performance of different construction heuristics (k=0 relaxation) on the MD-GmTSP. The construction phase allowed angle optimization only for new unvisited targets to be inserted into tours while keeping the rest of the tour unchanged (k=0). The initial feasible solutions were generated without imposing any time limit. The results demonstrate that the cheapest construction method produced min-max tours with an average cost that was 11.81% higher than the nearest construction method. However, the nearest and farthest construction methods produced tours with identical min-max costs. Additionally, the cheapest-insertion heuristics generated solutions approximately 2.5 times faster than the nearest-insertion method.

#### 7.2.2. Analysis of VNS Improvements for MD-GmTSP

**Figure 7:**
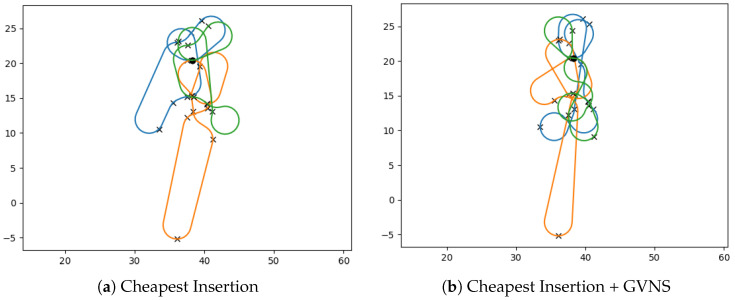

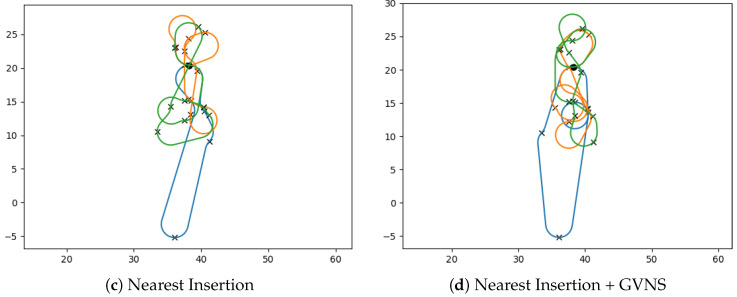
Feasible MD-GmTSP paths for the Ulysses22 instance for m=3 vehicles and h=8 angle discretizations obtained from different tour construction and GVNS heuristics. The vehicle paths are differentiated by the colors of the paths.

The impact of different neighborhood structures on improving the MD-GmTSP tours using BVNS, VND, and GVNS heuristics is shown in Table 3, Table 4 and Table 5, respectively. These improvement heuristics were executed with a computational time limit of $t_{max}=600$ s (10 min) as the stopping criterion, with FirstImprovement used to switch between neighborhoods. The presented results in each row of Table 3, Table 4 and Table 5 represent the average MD-GmTSP solutions for all TSPLIB instances considered. The analysis reveals that the improvement heuristics can achieve solution quality improvements of up to 30.78%. Furthermore, the average time required to solve these instances ranged from 898.6 s to 1439.96 s. Refer to Figure 7 for sample solutions with respect to the GVNS heuristic.

**Figure 8:**
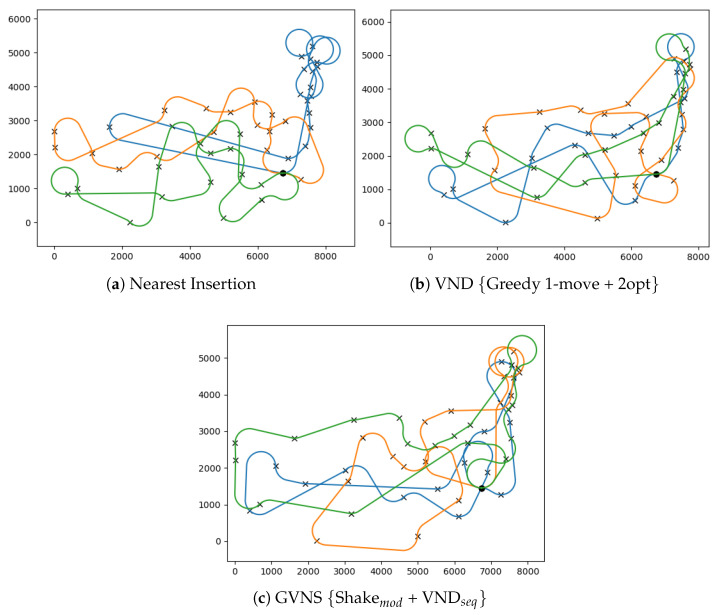
Feasible MD-GmTSP paths for the att48 instance for m=3 vehicles and h=8 angle discretizations obtained from tour construction and improvement heuristics. The vehicle paths are differentiated by the colors of the paths.

Table 6 presents the best-performing combination of construction and improvement heuristics for each instance. The most effective results are achieved using cheapest insertion and nearest insertion as the construction heuristics, in addition to GVNS with Shakemod and VND with one-point move as the improvement heuristics. Refer to Figure 8 for sample solutions corresponding to the att48 instance. It should be noted that solutions generated using the cheapest-insert construction with VNS have, on average, costs that are 14.96% higher than the solutions produced by the nearest-insert construction with VNS. On the other hand, the GVNS + Shake${}_{mod}$ scheme resulted in an average improvement of 3.46% over the initial tours, while the VND + one-point move scheme generated an average improvement of 3.32% over the initial solution. A comparison is made between the best solutions obtained from VNS-based heuristics and MILP formulations for each instance in Table 7. It is observed that VNS heuristics generally produce superior solutions in significantly less time than the MILP solutions obtained from CPLEX.

### 7.3. Learning-Based Approach

**Figure 9:**
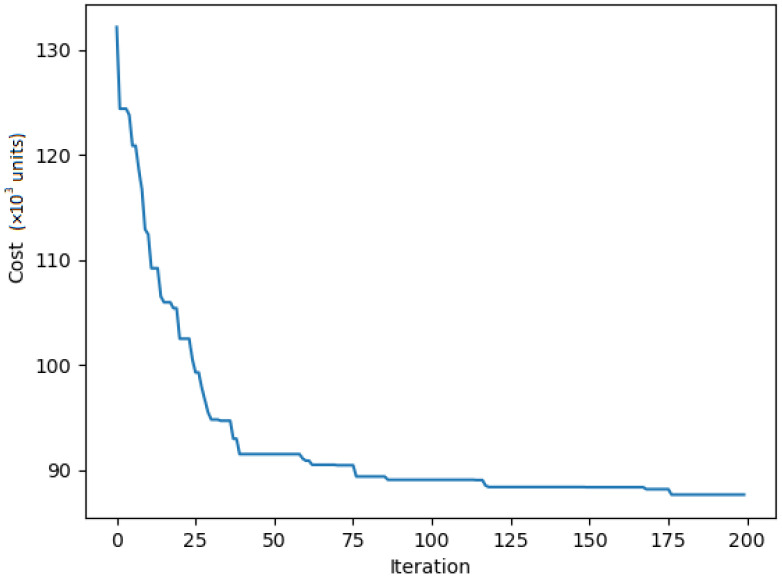
Learning curve for Dubins pr 76.

In the learning-based approach, training and testing datasets were generated separately for *m* Dubins agents and *n* cities. Each city coordinate was uniformly generated at random in the interval ${\left(0,1\right)}^{2}$ and paired with h=8 heading angles in the range [0,2π]. During training, the data were randomly generated at every iteration. To overcome performance limitations, separate models were trained for different numbers of cities and radius of curvature (ρ) specific to each instance. A total of 1000 instances were generated for each type and utilized to train the RL model described in Section 6. The RL model was trained using the Adam optimization method with the following hyperparameters: a learning rate of =$1e^{-5}$, a clipping gradients norm of 3, and S set of 10. Figure 9 illustrates the convergence of a model trained on the pr76 dataset after approximately 2000 iterations during the training process. The dataset consists of 1000 instances, each comprising 76 cities randomly distributed within the original boundaries of the pr76 instances. The tours for these instances are calculated using a Dubins turning radius (ρ=980).

In order to assess the effectiveness of the RL model, we compare its solutions to those generated by a heuristics-based approach for the MD-GmTSP. The trained RL model is utilized to evaluate the min-max tours for the original pr76 instance, ensuring a valid basis for comparison between the two approaches. Table 8 compares the solutions obtained from the RL model to the best solutions obtained from the MILP and VNS-based formulation. It is important to note that due to the absence of pre-existing baselines for the Dubins MTSP, it was not possible to determine the optimality gap for the solutions obtained from the RL model.

It can be observed that the RL model outperformed both the MILP formulation and the VNS-based heuristics on smaller instances of the problem. Specifically, the RL-based solutions performed better in terms of solution quality for smaller instances. However, as the problem instances became larger, the VNS-based heuristics generated superior solutions compared to the RL model. This indicates that the RL model is especially effective for solving MD-GmTSP instances with a relatively small number of cities and Dubins agents. This effectiveness can be attributed to the fact that the quality of Dubins tours used during the training phase is higher for smaller instances, while the training data quality decreases with the increase in instance size. The RL model’s ability to learn and generalize patterns from the training data enables it to achieve high-quality solutions in these instances. There is scope for additional research and experimentation to explore the potential for enhancing the RL model performance and scalability for larger instances of the MD-GmTSP by leveraging advanced RL techniques and alternative network architectures.

## 8. Conclusions

In this paper, we explore different methods to solve MinMax routing problems for a team of Dubins vehicles. We formulate the routing problem as a one-in-a-set mTSP with Dubins paths and refer to it as the MD-GmTSP. We then develop a MILP formulation for the MD-SmTSP and solve it on 16 TSPLIB instances (ranging from 16 to 137 cities) using CPLEX for finding optimal solutions. We show that this method does not scale well for large instances involving higher numbers of nodes or robots. As an alternate approach, we develop fast heuristics based on insertion techniques to obtain good, feasible solutions for the MD-GmTSP. The results show that, on average, the nearest-insertion algorithm generates solutions with tour costs 11.81% lower than the solutions constructed using the cheapest-insertion algorithm. Subsequently, a combination of VNS-based heuristics with several neighborhood search structures is explored to improve the quality of initial feasible solution within a 600 s time limit. The neighborhood structures we studied include 2-opt, 3-opt, k-point, Or-opt, Shake, and the Shake${}_{mod}$. We solved the TSPLIB instances to check the performance of different combinations of heuristics and identified the best combination to solve MD-GmTSP. Overall, GVNS-based heuristics produced the most promising improvements over the initial solutions. The best-performing combination to solve the MD-GmTSP was to use the nearest-insertion heuristic to construct the initial tour followed by a GVNS-type heuristic to improve the solution quality using neighborhood search structures. Finally, we also explore a learning-based method to generate solutions for the MD-GmTSP. Our architecture consists of a shared graph neural network, with distributed policy networks that extract a common policy for the Dubins multiple traveling salesmen. An S-sample batch reinforcement learning method trains the model, achieving significant improvements in convergence speed and performance. The resulting RL model is used to generate fast feasible paths to the MD-GmTSP, and a comprehensive comparison is presented on the solution quality obtained from each of the other approaches. Overall, the learning based methods work well for smaller instances, while the GVNS based approaches perform better for large instances. Future work can address factors related to planning paths in the presence of obstacles and uncertainties in the position of targets.

## Figures and Tables

**Table 1:** MD-GmTSP results for m=3 vehicles and h=8 discretizations on TSPLIB instances using CPLEX 12.10 with a runtime of 3 h (10,800 s). *Instances that CPLEX could not solve are indicated by* *.

Instance	Targets	Turning Radius (ρ)	Minmax Cost	Optimality Gap (%)
att48	47	394	28,315.3486	42.23
berlin52	51	55	5433.474	43.54
ch130 *	129	39	-	-
eil101 *	100	4	-	-
eil51	50	4	333.7285	46.46
eil76 *	75	4	-	-
gr137 *	136	4	-	-
gr96 *	95	4	-	-
kroA100 *	99	200	-	-
pr136 *	135	516	-	-
pr76 *	75	980	-	-
rat99 *	98	5	-	-
rd100 *	99	57	-	-
st70 *	69	6	-	-
ulysses16	15	2	57.7487	19.27
ulysses22	21	2	58.0386	14.09

**Table 2:** Comparison of MinMax costs of feasible solutions obtained from insertion heuristics for the MD-GmTSP for m=3 vehicles, C=8 angle discretizations, and k=0 relaxation. Times are specified in secs.

Instance	Radius	Cheapest Insertion		Farthest Insertion		Nearest Insertion		$\frac{c_{c}}{c_{n}}$ (%)
		Cost ($c_{c}$)	Time ($t_{c}$)	Cost ($c_{f}$)	Time ($t_{f}$)	Cost ($c_{n}$)	Time ($t_{n}$)	
att48	394	32,336.39	43.38	28,197.11	108.72	28,197.11	107.47	114.68
berlin52	55	6025.99	54.05	5453.38	138.37	5453.38	138.63	110.50
ch130	39	6472.87	751.58	5998.74	1904.17	5998.74	1944.28	107.90
eil101	4	554.77	361.14	450.26	898.30	450.26	900.88	123.21
eil51	4	308.30	50.70	277.73	126.92	277.73	128.76	111.01
eil76	4	416.51	157.05	345.66	394.27	345.66	402.35	120.50
gr137	4	847.06	850.68	701.39	2154.25	701.39	2217.90	120.77
gr96	4	551.00	311.42	488.93	770.56	488.93	780.32	112.69
kroA100	200	27,236.35	355.99	22,592.95	872.53	22,592.95	879.44	120.55
pr136	516	98,637.39	842.15	82,162.29	2130.30	82,162.29	2191.75	120.05
pr76	980	97,434.78	156.94	99,023.43	398.98	99,023.43	400.01	98.40
rat99	5	798.94	337.41	819.30	841.77	819.30	850.65	97.51
rd100	57	8679.56	343.93	6833.40	870.01	6833.40	869.33	127.02
st70	6	593.76	125.90	550.76	310.80	550.76	318.95	107.81
ulysses16	2	66.40	2.57	76.34	5.50	76.34	5.94	86.97
ulysses22	2	88.00	5.26	80.49	12.78	80.49	12.94	109.33

**Table 3:** Influence of different neighborhood structures in the BVNS heuristic on improving the MD-GmTSP solutions with $t_{max}=600$ s time limit.

Construction Heuristic	$N_{1}$		$N_{2}$	Average Improvement (%)	Max Improvement (%)	Standard Deviation	Average Computation Time (s)
	Move	Insertion	Local Search				
Cheapest	Shake	Greedy	2-opt	3.201	21.627	6.225	900.413
Cheapest	Shake	Greedy	3-opt	2.750	19.456	5.648	909.045
Cheapest	Shake	Random	2-opt	1.987	17.399	5.309	899.961
Cheapest	Shake	Random	3-opt	2.091	17.082	5.244	907.608
Cheapest	Shake${}_{mod}$	Greedy	2-opt	2.802	15.755	5.392	900.571
Cheapest	Shake${}_{mod}$	Greedy	3-opt	2.423	13.508	4.017	916.210
Cheapest	Shake${}_{mod}$	Random	2-opt	1.823	14.484	4.640	900.069
Cheapest	Shake${}_{mod}$	Random	3-opt	1.938	14.484	3.921	919.457
Farthest	Shake	Greedy	2-opt	2.785	24.736	7.609	1354.735
Farthest	Shake	Greedy	3-opt	2.585	25.342	6.969	1381.107
Farthest	Shake	Random	2-opt	1.703	18.939	5.043	1359.844
Farthest	Shake	Random	3-opt	1.650	20.988	5.331	1371.328
Farthest	Shake${}_{mod}$	Greedy	2-opt	2.526	26.589	6.931	1366.066
Farthest	Shake${}_{mod}$	Greedy	3-opt	1.986	26.567	6.598	1374.865
Farthest	Shake${}_{mod}$	Random	2-opt	1.470	21.869	5.455	1362.314
Farthest	Shake${}_{mod}$	Random	3-opt	1.135	18.158	4.540	1373.770
Nearest	Shake	Greedy	2-opt	2.750	25.154	7.015	1355.997
Nearest	Shake	Greedy	3-opt	2.550	25.025	6.781	1376.476
Nearest	Shake	Random	2-opt	1.745	20.988	5.415	1363.908
Nearest	Shake	Random	3-opt	1.896	20.988	5.600	1368.852
Nearest	Shake${}_{mod}$	Greedy	2-opt	2.627	26.589	6.932	1370.339
Nearest	Shake${}_{mod}$	Greedy	3-opt	1.635	26.142	6.535	1375.723
Nearest	Shake${}_{mod}$	Random	2-opt	1.617	20.351	5.173	1359.805
Nearest	Shake${}_{mod}$	Random	3-opt	1.477	16.364	4.335	1378.464

**Table 4:** Influence of different neighborhood structures in VND heuristic on improving the MD-GmTSP tours with $t_{max}=600$ s time limit.

Construction Heuristic	$N_{1}$		$N_{2}$	Average Improvement (%)	Max Improvement (%)	Standard Deviation	Average Computation Time (s)
	Move	Insertion	Local Search				
Cheapest	2-move	Greedy	2-opt	4.461	26.670	7.283	899.159
Cheapest	2-move	Greedy	3-opt	3.377	21.675	6.248	912.802
Cheapest	2-move	Random	2-opt	2.191	18.315	5.487	898.602
Cheapest	2-move	Random	3-opt	1.912	16.786	5.201	916.977
Cheapest	1-move	Greedy	2-opt	5.644	29.894	8.035	899.059
Cheapest	1-move	Greedy	3-opt	4.603	26.105	7.240	914.916
Cheapest	1-move	Random	2-opt	2.547	19.881	5.932	899.179
Cheapest	1-move	Random	3-opt	2.596	17.557	5.607	913.728
Farthest	2-move	Greedy	2-opt	3.483	25.342	7.802	1372.255
Farthest	2-move	Greedy	3-opt	2.997	25.202	6.868	1373.696
Farthest	2-move	Random	2-opt	1.922	20.988	5.633	1365.133
Farthest	2-move	Random	3-opt	1.958	20.988	5.514	1381.819
Farthest	1-move	Greedy	2-opt	3.729	25.622	8.050	1356.863
Farthest	1-move	Greedy	3-opt	3.691	25.622	7.486	1374.722
Farthest	1-move	Random	2-opt	2.200	23.350	6.111	1358.016
Farthest	1-move	Random	3-opt	2.180	23.913	6.225	1376.558
Nearest	2-move	Greedy	2-opt	3.462	25.342	7.598	1358.205
Nearest	2-move	Greedy	3-opt	3.181	25.342	7.029	1374.512
Nearest	2-move	Random	2-opt	1.775	20.988	5.447	1362.595
Nearest	2-move	Random	3-opt	1.822	20.988	5.502	1379.830
Nearest	1-move	Greedy	2-opt	4.494	26.589	8.430	1360.322
Nearest	1-move	Greedy	3-opt	4.167	25.622	7.535	1372.429
Nearest	1-move	Random	2-opt	2.068	23.990	6.232	1367.803
Nearest	1-move	Random	3-opt	1.855	23.350	5.911	1375.266

**Table 5:** Influence of different neighborhood structures in GVNS on improving the MD-GmTSP tours with $t_{max}=600$ s time limit.

Construction Heuristic	$N_{1}$		$N_{2}$	Average Improvement (%)	Max Improvement (%)	Standard Deviation	Average Computation Time (s)
	Move	Insertion	Local Search				
Cheapest	Shake	Greedy	VND${}_{seq}$	5.862	22.840	6.842	915.206
Cheapest	Shake	Random	VND${}_{seq}$	2.241	21.121	6.233	969.554
Cheapest	Shake${}_{mod}$	Greedy	VND${}_{seq}$	6.165	23.141	7.486	919.908
Cheapest	Shake${}_{mod}$	Random	VND${}_{seq}$	2.902	21.666	6.005	974.873
Farthest	Shake	Greedy	VND${}_{seq}$	2.595	26.589	7.208	1370.046
Farthest	Shake	Random	VND${}_{seq}$	2.399	26.589	7.089	1406.246
Farthest	Shake${}_{mod}$	Greedy	VND${}_{seq}$	2.996	26.589	7.226	1368.846
Farthest	Shake${}_{mod}$	Random	VND${}_{seq}$	2.813	25.548	7.052	1409.714
Nearest	Shake	Greedy	VND${}_{seq}$	3.555	24.363	7.183	1368.566
Nearest	Shake	Random	VND${}_{seq}$	2.176	23.506	6.349	1412.890
Nearest	Shake${}_{mod}$	Greedy	VND${}_{seq}$	4.222	25.799	7.535	1391.104
Nearest	Shake${}_{mod}$	Random	VND${}_{seq}$	2.078	23.170	6.161	1433.516

**Table 6:** Best-performing combination of heuristics for the MD-GmTSP. Time is specified in secs.

Instance	Cities	Radius	Construction Phase			Improvement Phase						Improvement (%)
			Heuristic	Cost	Time	Scheme	$N_{1}$		$N_{2}$	Cost	Time	
							Move	Insertion	Move			
att48	48	394	Cheapest	32,336.39	43.90	GVNS	Shake${}_{mod}$	Greedy	VND${}_{Seq}$	25,775.29	646.83	20.29
berlin52	52	55	Nearest	5453.38	139.50	GVNS	Shake	Greedy	VND${}_{Seq}$	5201.58	744.59	4.62
ch130	130	39	Nearest	5998.74	1931.72	VND	1-point	Greedy	2-opt	5839.33	2533.20	2.66
eil101	101	4	Nearest	450.26	901.52	VND	2-point	Greedy	2-opt	410.15	1502.13	8.91
eil51	51	4	Nearest	277.73	131.30	VND	1-point	Greedy	2-opt	274.60	731.93	1.13
eil76	76	4	Nearest	345.66	397.13	VND	1-point	Greedy	3-opt	344.29	1016.68	0.40
gr137	137	4	Nearest	701.39	2196.77	GVNS	Shake${}_{mod}$	Greedy	VND${}_{Seq}$	668.91	2939.97	4.63
gr96	96	4	Nearest	488.93	782.38	VND	1-point	Greedy	3-opt	457.93	1401.15	6.34
kroA100	100	200	Nearest	22,592.95	904.13	GVNS	Shake${}_{mod}$	Greedy	VND${}_{Seq}$	22,321.63	1536.18	1.20
pr136	136	516	Farthest	82,162.29	2180.81	GVNS	Shake${}_{mod}$	Greedy	VND${}_{Seq}$	81,379.62	2786.21	0.95
pr76	76	980	Nearest	99,023.43	393.95	GVNS	Shake${}_{mod}$	Greedy	VND${}_{Seq}$	90,809.31	997.12	8.30
rat99	99	5	Cheapest	798.94	334.75	VND	1-point	Greedy	3-opt	787.09	952.94	1.48
rd100	100	57	Nearest	6833.40	867.27	GVNS	Shake${}_{mod}$	Greedy	VND${}_{Seq}$	6827.48	1472.39	0.09
st70	70	6	Farthest	550.76	318.94	VND	1-point	Greedy	3-opt	550.75	951.81	0.00
ulysses16	16	2	Cheapest	66.40	2.24	GVNS	Shake${}_{mod}$	Greedy	VND${}_{Seq}$	56.04	614.09	15.60
ulysses22	22	2	Nearest	80.49	12.98	VND	1-point	Greedy	2-opt	60.31	613.11	25.08

**Table 7:** Comparison for best solutions obtained from MILP formulation ($t_{max}=$ 10,800 s) against the VNS-based heuristics ($t_{max}=600$ s).

Instance	Cities	Radius	VNS Solution		MILP Solution		$\frac{C_{m}-C_{h}}{C_{h}}*100$
			Cost ($C_{h}$)	Time ($T_{h}$)	Cost ($C_{m}$)	Optimality Gap	
att48	48	394	25,775.29	690.72	28,315.35	42.23	9.85
berlin52	52	55	5201.58	884.09	5433.47	43.54	4.46
ch130	130	39	5839.33	4464.92	-	-	-
eil101	101	4	410.15	2403.65	-	-	-
eil51	51	4	274.60	863.23	333.73	46.46	21.53
eil76	76	4	344.29	1413.81	-	-	-
gr137	137	4	668.91	5136.74	-	-	-
gr96	96	4	457.93	2183.53	-	-	-
kroA100	100	200	22,321.63	2440.30	-	-	-
pr136	136	516	81,379.62	4967.02	-	-	-
pr76	76	980	90,809.31	1391.07	-	-	-
rat99	99	5	787.09	1287.69	-	-	-
rd100	100	57	6827.48	2339.65	-	-	-
st70	70	6	550.75	1270.75	-	-	-
ulysses16	16	2	56.04	616.33	57.75	19.27	3.05
ulysses22	22	2	60.31	626.09	58.04	14.09	−3.76

**Table 8:** Comparison for best solutions obtained from MILP formulation ($t_{max}=$ 10,800 s) against the VNS-based heuristics ($t_{max}=600$ s).

Instance	Cities	Radius	VNS Solution	MILP Solution		RL Solution	
			Cost ($C_{h}$)	Cost ($C_{m}$)	$\frac{C_{m}}{C_{h}}$	Cost ($C_{RL}$)	$\frac{C_{RL}}{C_{h}}$
att48	48	394	25,775.29	28,315.35	1.10	22,846.38	0.89
eil51	51	4	274.60	333.73	1.22	252.64	0.92
pr76	76	980	90,809.31	-	-	98,174.43	1.08
rd100	100	57	6827.48	-	-	7549.84	1.11
st70	70	6	550.75	-	-	564.64	1.03
ulysses16	16	2	56.04	57.75	1.03	54.86	0.98
ulysses22	22	2	60.31	58.04	0.96	57.98	0.96

## Data Availability

The data used in this study are openly available at http://comopt.ifi.uni-heidelberg.de/software/TSPLIB95/. Refer to [40].

## References

[1] Lawler E.L., Lenstra J.K., Rinnooy Kan A.H., Shmoys D.B. (1986). Erratum: The traveling salesman problem: A guided tour of combinatorial optimization. J. Oper. Res. Soc..

[2] Laporte G. (1992). The traveling salesman problem: An overview of exact and approximate algorithms. Eur. J. Oper. Res..

[3] Boscariol P., Gasparetto A., Scalera L., Carbone G., Laribi M.A. (2023). Path Planning for Special Robotic Operations. Robot Design: From Theory to Service Applications.

[4] Ham A.M. (2018). Integrated scheduling of m-truck, m-drone, and m-depot constrained by time-window, drop-pickup, and m-visit using constraint programming. Transp. Res. Part C Emerg. Technol..

[5] Venkatachalam S., Sundar K., Rathinam S. (2018). A two-stage approach for routing multiple unmanned aerial vehicles with stochastic fuel consumption. Sensors.

[6] Cheikhrouhou O., Koubâa A., Zarrad A. (2020). A cloud based disaster management system. J. Sens. Actuator Netw..

[7] Bähnemann R., Lawrance N., Chung J.J., Pantic M., Siegwart R., Nieto J., Ishigami G., Yoshida K. (2021). Revisiting Boustrophedon Coverage Path Planning as a Generalized Traveling Salesman Problem. Field and Service Robotics.

[8] Conesa-Muñoz J., Pajares G., Ribeiro A. (2016). Mix-opt: A new route operator for optimal coverage path planning for a fleet in an agricultural environment. Expert Syst. Appl..

[9] Zhao W., Meng Q., Chung P.W. (2015). A heuristic distributed task allocation method for multivehicle multitask problems and its application to search and rescue scenario. IEEE Trans. Cybern..

[10] Xie J., Carrillo L.R.G., Jin L. (2019). An Integrated Traveling Salesman and Coverage Path Planning Problem for Unmanned Aircraft Systems. IEEE Control Syst. Lett..

[11] Hari S.K.K., Nayak A., Rathinam S. (2020). An Approximation Algorithm for a Task Allocation, Sequencing and Scheduling Problem Involving a Human-Robot Team. IEEE Robot. Autom. Lett..

[12] Gorenstein S. (1970). Printing press scheduling for multi-edition periodicals. Manag. Sci..

[13] Saleh H.A., Chelouah R. (2004). The design of the global navigation satellite system surveying networks using genetic algorithms. Eng. Appl. Artif. Intell..

[14] Angel R., Caudle W., Noonan R., Whinston A. (1972). Computer-assisted school bus scheduling. Manag. Sci..

[15] Brumitt B.L., Stentz A. Dynamic mission planning for multiple mobile robots. Proceedings of the IEEE International Conference on Robotics and Automation.

[16] Yu Z., Jinhai L., Guochang G., Rubo Z., Haiyan Y. An implementation of evolutionary computation for path planning of cooperative mobile robots. Proceedings of the 4th World Congress on Intelligent Control and Automation (Cat. No. 02EX527).

[17] Ryan J.L., Bailey T.G., Moore J.T., Carlton W.B. Reactive tabu search in unmanned aerial reconnaissance simulations. Proceedings of the 1998 Winter Simulation Conference. Proceedings (Cat. No. 98CH36274).

[18] Dubins L.E. (1957). On curves of minimal length with a constraint on average curvature, and with prescribed initial and terminal positions and tangents. Am. J. Math..

[19] Reeds J., Shepp L. (1990). Optimal paths for a car that goes both forwards and backwards. Pac. J. Math..

[20] Sussmann H.J., Tang G. (1991). Shortest paths for the Reeds-Shepp car: A worked out example of the use of geometric techniques in nonlinear optimal control. Rutgers Cent. Syst. Control Tech. Rep..

[21] Boissonnat J.D., Cérézo A., Leblond J. (1994). Shortest paths of bounded curvature in the plane. J. Intell. Robot. Syst..

[22] Kolmogorov A.N., Mishchenko Y.F., Pontryagin L.S. (1962). A Probability Problem of Optimal Control.

[23] Tang Z., Ozguner U. (2005). Motion planning for multitarget surveillance with mobile sensor agents. IEEE Trans. Robot..

[24] Rathinam S., Sengupta R., Darbha S. (2007). A Resource Allocation Algorithm for Multivehicle Systems with Nonholonomic Constraints. IEEE Trans. Autom. Sci. Eng..

[25] LaValle S.M. (2006). Planning Algorithms.

[26] Ny J., Feron E., Frazzoli E. (2011). On the Dubins traveling salesman problem. IEEE Trans. Autom. Control.

[27] Manyam S.G., Rathinam S., Darbha S., Obermeyer K.J. (2015). Lower bounds for a vehicle routing problem with motion constraints. Int. J. Robot. Autom.

[28] Ma X., Castanon D.A. Receding horizon planning for Dubins traveling salesman problems. Proceedings of the 45th IEEE Conference on Decision and Control.

[29] Savla K., Frazzoli E., Bullo F. (2008). Traveling salesperson problems for the Dubins vehicle. IEEE Trans. Autom. Control.

[30] Yadlapalli S., Malik W., Darbha S., Pachter M. (2009). A Lagrangian-based algorithm for a multiple depot, multiple traveling salesmen problem. Nonlinear Anal. Real World Appl..

[31] Macharet D.G., Campos M.F. (2014). An orientation assignment heuristic to the Dubins traveling salesman problem. Proceedings of the Ibero-American Conference on Artificial Intelligence, Santiago de.

[32] Sujit P., Hudzietz B., Saripalli S. (2013). Route planning for angle constrained terrain mapping using an unmanned aerial vehicle. J. Intell. Robot. Syst..

[33] Isaiah P., Shima T. (2015). Motion planning algorithms for the Dubins travelling salesperson problem. Automatica.

[34] Babel L. (2020). New heuristic algorithms for the Dubins traveling salesman problem. J. Heuristics.

[35] Manyam S.G., Rathinam S. (2018). On tightly bounding the dubins traveling salesman’s optimum. J. Dyn. Syst. Meas. Control.

[36] Manyam S.G., Rathinam S., Darbha S. (2015). Computation of lower bounds for a multiple depot, multiple vehicle routing problem with motion constraints. J. Dyn. Syst. Meas. Control.

[37] Cohen I., Epstein C., Shima T. (2017). On the discretized dubins traveling salesman problem. IISE Trans..

[38] Oberlin P., Rathinam S., Darbha S. (2010). Today’s traveling salesman problem. IEEE Robot. Autom. Mag..

[39] Hansen P., Mladenović N. (2001). Variable neighborhood search: Principles and applications. Eur. J. Oper. Res..

[40] Reinhelt G. (2014). {TSPLIB}: A Library of Sample Instances for the TSP (and Related Problems) from Various Sources and of Various Types. http://comopt.ifi.uni-heidelberg.de/software/TSPLIB95/.

[41] Applegate D.L., Bixby R.E., Chvatal V., Cook W.J. (2007). The Traveling Salesman Problem: A Computational Study (Princeton Series in Applied Mathematics).

[42] Vazirani V.V. (2001). Approximation Algorithms.

[43] Rosenkrantz D.J., Stearns R.E., Lewis P.M., Ravi S.S., Shukla S.K. (2009). An analysis of several heuristics for the traveling salesman problem. Fundamental Problems in Computing: Essays in Honor of Professor Daniel J. Rosenkrantz.

[44] Manyam S., Rathinam S., Casbeer D. Dubins paths through a sequence of points: Lower and upper bounds. Proceedings of the 2016 International Conference on Unmanned Aircraft Systems (ICUAS).

[45] Sundar K., Rathinam S. (2014). Algorithms for routing an unmanned aerial vehicle in the presence of refueling depots. IEEE Trans. Autom. Sci. Eng..

[46] Sundar K., Rathinam S. An exact algorithm for a heterogeneous, multiple depot, multiple traveling salesman problem. Proceedings of the 2015 International Conference on Unmanned Aircraft Systems.

[47] Sundar K., Venkatachalam S., Rathinam S. (2016). An Exact Algorithm for a Fuel-Constrained Autonomous Vehicle Path Planning Problem. arXiv.

[48] Lo K.M., Yi W.Y., Wong P.K., Leung K.S., Leung Y., Mak S.T. (2018). A genetic algorithm with new local operators for multiple traveling salesman problems. Int. J. Comput. Intell. Syst..

[49] Bao X., Wang G., Xu L., Wang Z. (2023). Solving the Min-Max Clustered Traveling Salesmen Problem Based on Genetic Algorithm. Biomimetics.

[50] Zhang Y., Han X., Dong Y., Xie J., Xie G., Xu X. (2021). A novel state transition simulated annealing algorithm for the multiple traveling salesmen problem. J. Supercomput..

[51] He P., Hao J.K. (2023). Memetic search for the minmax multiple traveling salesman problem with single and multiple depots. Eur. J. Oper. Res..

[52] He P., Hao J.K. (2022). Hybrid search with neighborhood reduction for the multiple traveling salesman problem. Comput. Oper. Res..

[53] Venkatesh P., Singh A. (2015). Two metaheuristic approaches for the multiple traveling salesperson problem. Appl. Soft Comput..

[54] Hamza A., Darwish A.H., Rihawi O. (2023). A New Local Search for the Bees Algorithm to Optimize Multiple Traveling Salesman Problem. Intell. Syst. Appl..

[55] Rathinam S., Rajagopal H. Optimizing Mission Times for Multiple Unmanned Vehicles with Vehicle-Target Assignment Constraints. Proceedings of the AIAA SCITECH 2022 Forum.

[56] Patil A., Bae J., Park M. (2022). An algorithm for task allocation and planning for a heterogeneous multi-robot system to minimize the last task completion time. Sensors.

[57] Dedeurwaerder B., Louis S.J. A Meta Heuristic Genetic Algorithm for Multi-Depot Routing in Autonomous Bridge Inspection. Proceedings of the 2022 IEEE Symposium Series on Computational Intelligence (SSCI).

[58] Park J., Kwon C., Park J. Learn to Solve the Min-max Multiple Traveling Salesmen Problem with Reinforcement Learning. Proceedings of the 2023 International Conference on Autonomous Agents and Multiagent Systems.

[59] Frederickson G.N., Hecht M.S., Kim C.E. Approximation algorithms for some routing problems. Proceedings of the 17th Annual Symposium on Foundations of Computer Science (sfcs 1976).

[60] Yadlapalli S., Rathinam S., Darbha S. (2010). 3-Approximation algorithm for a two depot, heterogeneous traveling salesman problem. Optim. Lett..

[61] Chour K., Rathinam S., Ravi R. S*: A Heuristic Information-Based Approximation Framework for Multi-Goal Path Finding. Proceedings of the International Conference on Automated Planning and Scheduling.

[62] Carlsson J.G., Ge D., Subramaniam A., Wu A. (2007). Solving Min-Max Multi-Depot Vehicle Routing Problem. Proceedings of the Lectures on Global Optimization (Volume 55 in the Series Fields Institute Communications).

[63] Venkata Narasimha K., Kivelevitch E., Sharma B., Kumar M. (2013). An ant colony optimization technique for solving Min-max Multi-Depot Vehicle Routing Problem. Swarm Evol. Comput..

[64] Lu L.C., Yue T.W. (2019). Mission-oriented ant-team ACO for min-max MTSP. Appl. Soft Comput..

[65] Liu J., Zhang Y., Wang X., Xu C., Ma X. Min-max Path Planning of Multiple UAVs for Autonomous Inspection. Proceedings of the 2020 International Conference on Wireless Communications and Signal Processing (WCSP).

[66] Wang X., Golden B.H., Wasil E.A. (2015). The min-max multi-depot vehicle routing problem: Heuristics and computational results. J. Oper. Res. Soc..

[67] Scott D., Manyam S.G., Casbeer D.W., Kumar M. Market Approach to Length Constrained Min-Max Multiple Depot Multiple Traveling Salesman Problem. Proceedings of the 2020 American Control Conference (ACC).

[68] Prasad A., Sundaram S., Choi H.L. Min-Max Tours for Task Allocation to Heterogeneous Agents. Proceedings of the 2018 IEEE Conference on Decision and Control (CDC).

[69] Banik S., Rathinam S., Sujit P. Min-Max Path Planning Algorithms for Heterogeneous, Autonomous Underwater Vehicles. Proceedings of the 2018 IEEE/OES Autonomous Underwater Vehicle Workshop (AUV).

[70] Ding L., Zhao D., Ma H., Wang H., Liu L. Energy-Efficient Min-Max Planning of Heterogeneous Tasks with Multiple UAVs. Proceedings of the 2018 IEEE 24th International Conference on Parallel and Distributed Systems (ICPADS).

[71] Deng L., Xu W., Liang W., Peng J., Zhou Y., Duan L., Das S.K. (2020). Approximation Algorithms for the Min-Max Cycle Cover Problem With Neighborhoods. IEEE/ACM Trans. Netw..

[72] Kara I., Bektas T. (2006). Integer linear programming formulations of multiple salesman problems and its variations. Eur. J. Oper. Res..

[73] Hansen P., Mladenović N., Pérez J.A.M. (2010). Variable neighbourhood search: Methods and applications. Ann. Oper. Res..

[74] Croes G.A. (1958). A method for solving traveling-salesman problems. Oper. Res..

[75] Lin S. (1965). Computer solutions of the traveling salesman problem. Bell Syst. Tech. J..

[76] Hopfield J.J., Tank D.W. (1985). “Neural” computation of decisions in optimization problems. Biol. Cybern..

[77] Vinyals O., Fortunato M., Jaitly N. (2015). Pointer networks. arXiv.

[78] Deudon M., Cournut P., Lacoste A., Adulyasak Y., Rousseau L.M. (2018). Learning heuristics for the tsp by policy gradient. Proceedings of the International Conference on the Integration of Constraint Programming, Artificial Intelligence, and Operations Research.

[79] Kool W., Van Hoof H., Welling M. (2018). Attention, learn to solve routing problems!. arXiv.

[80] Bello I., Pham H., Le Q.V., Norouzi M., Bengio S. (2016). Neural combinatorial optimization with reinforcement learning. arXiv.

[81] Nazari M., Oroojlooy A., Snyder L., Takác M. Reinforcement learning for solving the vehicle routing problem. Proceedings of the 32nd Annual Conference on Neural Information Processing Systems (NIPS 2018).

[82] Hu Y., Yao Y., Lee W.S. (2020). A reinforcement learning approach for optimizing multiple traveling salesman problems over graphs. Knowl.-Based Syst..

[83] Park J., Bakhtiyar S., Park J. (2021). ScheduleNet: Learn to solve multi-agent scheduling problems with reinforcement learning. arXiv.

[84] Xu K., Hu W., Leskovec J., Jegelka S. (2018). How powerful are graph neural networks?. arXiv.

[85] Rosenkrantz D.J., Stearns R.E., Lewis P.M. (1977). An analysis of several heuristics for the traveling salesman problem. SIAM J. Comput..

[86] Helsgaun K. (2000). An effective implementation of the Lin–Kernighan traveling salesman heuristic. Eur. J. Oper. Res..

[87] IBM ILOG CPLEX Optimizer (2012). En Ligne. https://www.ibm.com/products/ilog-cplex-optimization-studio/cplex-optimizer.

[88] Applegate D., Bixby R., Chvatal V., Cook W. (2006). Concorde TSP Solver. https://www.math.uwaterloo.ca/tsp/concorde.

[89] Helsgaun K. (2017). An Extension of the Lin-Kernighan-Helsgaun TSP Solver for Constrained Traveling Salesman and Vehicle Routing Problems.

[90] Vaswani A., Shazeer N., Parmar N., Uszkoreit J., Jones L., Gomez A.N., Kaiser Ł., Polosukhin I. Attention is all you need. In Proceedings of the 31st Annual Conference on Neural Information Processing Systems (NIPS 2017).

[91] Williams R.J. (1992). Simple statistical gradient-following algorithms for connectionist reinforcement learning. Mach. Learn..

[92] Walker A. (2020). pyDubins. https://github.com/AndrewWalker/pydubins.

